# Impact of a Specific Amino Acid Composition with Micronutrients on Well-Being in Subjects with Chronic Psychological Stress and Exhaustion Conditions: A Pilot Study

**DOI:** 10.3390/nu10050551

**Published:** 2018-04-29

**Authors:** Deborah Armborst, Christine Metzner, Birgit Alteheld, Norman Bitterlich, Daniela Rösler, Roswitha Siener

**Affiliations:** 1 Department of Urology, Medical Nutrition Science, University of Bonn, Sigmund-Freud Str. 25, D-53105 Bonn, Germany; Roswitha.Siener@ukbonn.de; 2 Bonn Education Association for Dietetics r. A., Fuerst-Pueckler-Str. 44, D-50935 Cologne, Germany; info-bfd@t-online.de or christine.metzner@rwth-aachen.de (C.M.); office@bfdev.de (D.R.); 3 Department of Internal Medicine III, Uniklinik RWTH Aachen, Pauwelsstr. 44, D-52074 Aachen, Germany; 4 Department of Nutrition and Food Sciences, Nutritional Physiology, University of Bonn, Nussallee 9, D-53115 Bonn, Germany; b.alteheld@uni-bonn.de; 5 Department of Biostatistics, Medicine and Service Ltd., Boettcherstr. 10, D-09117 Chemnitz, Germany; bitterlich@medizinservice-sachsen.de

**Keywords:** stress management, perceived stress questionnaire (PSQ_30_), dietary supplement, hypothalamus–pituitary–adrenal axis

## Abstract

Chronic work-life stress leads to dysfunction of the hypothalamus–pituitary–adrenal axis, the autonomic nervous system, and the serotonergic system, with resultant impairment of overall well-being. Aim of the study was to improve perceived stress by a specific amino acid composition with micronutrients in the verum versus placebo group. A total of 59 participants (18–65 years) with self-reported perceived chronic stress and exhaustion conditions participated in this randomized, double-blind, placebo-controlled study. The Perceived Stress Questionnaire (PSQ_30_), amino acid profile, anthropometric, clinical, blood, urine parameters, and dietary intake were assessed. After 12 weeks, the verum group achieved significantly greater improvements in the total PSQ_30_ score compared with the placebo group. In the verum group, serum taurine concentration, folic acid concentration, urinary magnesium excretion, and the ratio of l-tryptophan to the sum of competing amino acids rose significantly. In the placebo group, serum concentrations of serotonin, protein, and magnesium decreased significantly, whereas the cardiometabolic risk parameters body weight, body mass index, waist circumference, and waist-to-height ratio increased significantly. Compared with placebo, the verum supplementation resulted in a higher improvement in perceived stress. Beneficial effects on the serotonergic system and preventive effects on magnesium homeostasis and some cardiometabolic risk factors were supposed. Additional effects might be caused by the optimized food intake.

## 1. Introduction

Chronic psychosocial stress has adverse effects on the physical and mental health of individuals and organizational effectiveness. It is recognized as a major challenge worldwide [1,2,3]. In the short run, biological responses to stress promote adaptation, maintenance of homeostasis, and survival (“allostasis”) via neuroendocrine, cardiovascular, autonomic, immune, and metabolic systems [4,5]. However, chronic stress leads to long-term dysregulations in these systems (“allostatic load”) that can promote and exacerbate pathophysiology [4,5]. Individuals under prolonged response to chronic emotional and interpersonal stressors and insufficient recovery are at high risk of developing long-term exhaustion conditions, such as burnout [6]. This occupationally specific dysphoria is characterized by emotional exhaustion, depersonalization, and reduced personal accomplishment [7,8]. The frequently debated question is whether burnout is a form of depression or a distinct phenomenon [9,10,11]. To date, burnout is not officially recognized as a mental disorder, and no generally accepted definition or diagnostic criteria exist [12,13].

The brain is the key organ for the stress response. Exposure to stress causes the activation of the sympathetic–adrenal–medullary (SAM) axis, resulting in the release of epinephrine and norepinephrine, which affect the heart rate and other autonomic changes, as well as the hypothalamus–pituitary–adrenal (HPA) axis, leading to the release of glucocorticoids such as cortisol [14,15]. During chronic stress, prolonged and repeated activation of the HPA axis occurs as an adaptive mechanism to stress [16]. This increased HPA axis activation can result in hypercortisolism. High cortisol concentrations play an important role in various psychoneuroendocrinological processes and hypertension. Insulin resistance, hyperglycemia, inflammation, visceral fat accumulation, and the metabolic syndrome are the consequences [17,18,19,20]. A state of hypofunctioning with related hypocortisolism can follow. To date, there is no consistent evidence of the occurrence of hypocortisolism in a clinical burnout patient group as a whole, but hypocortisolism is discussed to be present in patients with more severe burnout symptoms [21]. Furthermore, chronic stress leads to hyperactivity of the sympathetic nervous system [15]. Possible related risks include increased heart rate, activation of the renin–angiotensin system, and oxidative stress [22].

The mesolimbic dopaminergic system and other brain regions involved in stress/motivation circuits may play important roles in chronic stress-induced food intake, food preference and reward sensitivity [23]. However, uncontrollable chronic stress exposure can lead to poor dietary choices, weight gain, diet-related metabolic risk [24], and increased specific nutrient requirements. An adequate supply of specific amino acids, as a precursor for neurotransmitters, is suggested to positively influence the stress-induced imbalance between excitatory (e.g., norepinephrine, dopamine, and glutamate) and inhibitory (e.g., serotonin, gamma-aminobutyric acid (GABA), and glycine) neurotransmitters and neurological, emotional and behavioral consequences [25]. Consuming the amino acid tyrosine, a precursor for dopamine and norepinephrine, is considered an effective cognition enhancer, when neurotransmitter function is intact and dopamine and/or norepinephrine are temporarily depleted [26]. Taurine plays a role in the central nervous system, in antioxidant and anti-inflammatory actions, and in several metabolic processes. Taurine acts as an inhibitory neuromodulator, as a cytoprotectant against stress-related neuronal damage, and is commonly known for its claimed energizing properties and anti-fatigue compound [27,28]. Low brain serotonin concentrations are associated with poor memory and depressed mood [29]. Cerebral serotonin is synthesized from l-tryptophan (l-Trp) with the rate limiting step being catalyzed by the enzyme tryptophan hydroxylase. The precursor l-Trp is transported across the blood–brain barrier. Several amino acids, the branched-chain amino acids (BCAA, such as l-leucine, l-isoleucine, and l-valine), and the aromatic l-tyrosine and l-phenylalanine, collectively known as the competing amino acids (CAA), are transported via the same carrier system [30]. Studies have shown that an increased plasma ratio of free l-Trp to the sum of CAA results in an uptake of l-Trp by the brain, suggesting that this condition may increase the synthesis of serotonin [30,31]. l-ornithine supplementation has shown potential to relieve stress, reduce HPA axis activity, and improve fatigue-related sleep quality [32]. Moreover, water-soluble vitamins, particularly B-vitamins and vitamin C, together with minerals, such as magnesium and zinc, act as cofactors in the synthesis and metabolism of neurotransmitters. Therefore, these micronutrients are essential for regulating the stress response [33,34]. An adequate intake of all eight B vitamins is essential for optimal physiological and neurological functioning. Folate, B_6_, and B_12_ are directly involved in neurotransmitter synthesis and homocysteine metabolism [35,36]. Vitamin C, a key antioxidant of the central nervous system, is involved in neuromodulation, -protection, and -transmission [37,38]. Zinc and magnesium are essential for adequate functioning of the neurotransmitter systems and exhibit antidepressant properties [39]. Individuals who are exposed to occupational pressures and a stressful lifestyle are at risk of marginal deficiencies of one or more of these micronutrients [33]. A previous meta-analysis concluded the beneficial effects of multivitamin/mineral supplementation on perceived stress, mild psychiatric symptoms, and aspects of everyday mood in apparently healthy individuals, but optimal levels of intake, optimal doses, and active ingredients were questionable [40].

The aim of this randomized, placebo-controlled, double-blind clinical trial was to evaluate the impact of a daily supplementation with a specific amino acid composition (taurine, l-ornithine, l-phenylalanine, and l-tyrosine) with micronutrients (vitamin C, seven B-vitamins and 5 minerals) on perceived stress (primary outcome), frequency of neurovegetative symptoms, and endocrine–cardiometabolic risk factors in women and men with chronic psychological stress and exhaustion conditions. The hypothesis of the study was that the verum supplementation results in a lower total Perceived Stress Questionnaire (PSQ_30_) score in participants with chronic stress and exhaustion conditions as compared with the placebo supplementation after 12 weeks.

## 2. Materials and Methods

### 2.1. Questionnaires: The PSQ_30_, the Psychological Neurological Questionnaire (PNF), and the Visual Analogue Scales (VAS)

The PSQ_30_ is a valid instrument to assess the extent of a subjectively perceived stress in the context of a transactional view of stress and reflects general strong psychometric properties [41,42]. The original PSQ includes 30 items, which are assigned to seven scales (harassment, overload, irritability, lack of joy, fatigue, worries, and tension). The items were answered with a four-point rating scale (1 = almost never, 2 = sometimes, 3 = often, and 4 = usually). All items are applicable to adults of any stage of life, sex, or occupation, but interpretable as specific to a variety of real-life-situations. Moreover, each item is worded in such a way that the neutral, cognitive aspect of experience is emphasized. Mean values for the seven scales were calculated. The total score was derived from the raw item scores that were linearly transformed to values between 0 (lowest possible level of stress) and 1 (highest possible level of stress).

The PNF includes 38 items about the frequency of neurovegetative symptoms within the past three months. The items are divided into five categories: psychoneurovegetative stability, neurological symptoms, impulsion, excitability, concentration, and memory [43]. The self-reported symptoms were assessed as total points and for individual categories, ranging from “not at all” (0 points) to “often” (3 points). For detailed information on the psychometric properties of the PNF refer to Schneider et al. [43].

The VAS was developed for this study on the basis of a Likert-type scale. This VAS was used to rate the subjective intensity of sensation by assessing the emotional states of avolition, fatigue, capability, mood swing, and indifference. The VAS consisted of a line whose endpoints were designated as “no sensation (1 point)” and “the most intense sensation imaginable (5 points).” In addition, five cartoon faces from happy face to sad face depicted 1–5 points. The participants were asked to mark the score on points 1–5 or the associated face on the line how they currently felt. The points of each line were summed up to the total VAS points. The psychometric properties of the VAS and Likert-type scale are specified by Hasson and Arnetz [44].

### 2.2. Participants

**Figure 1:**
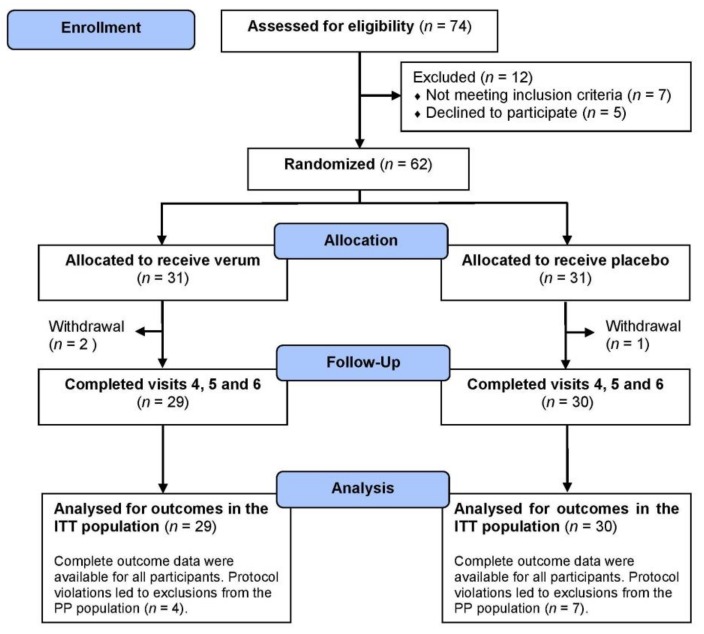
Flow diagram of participants. Explanations: visit 1: assessed for eligibility; from visit 2 to visit 3: run-in period; visit 3: baseline/randomization; visit 4: after four weeks; visit 5: after eight weeks; visit 6: after 12 weeks. In the verum group, one subject was excluded due to abnormal blood values at baseline. Further reasons for withdrawal are explained in the results below. Protocol violations: The 12-week intervention phase was planned with a tolerance range of 84 ± 2 days. Because of private reasons, a few subjects could not meet the visit 6 in this time range. Thus, the last visit took place a few days earlier (verum and placebo group: *n* = 2) or later (verum group: *n* = 1; placebo group: *n* = 2) and consequently, under- or over-consumption of the verum or placebo treatment was mostly accompanied. Moreover, there was an over-consumption in the verum group (*n* = 1) and in the placebo group (*n* = 3). Abbreviations: ITT, intention to treat; PP, per protocol.

Participants with subjectively perceived chronic stress and exhaustion conditions, such as symptoms of burnout, were recruited via local media and online advertising, internal mail systems, flyers and printed media distributed in the Cologne/Bonn region, Germany. Perceived chronic psychological stress was defined by an elevated level of the standardized PSQ_30_ [42]. At the beginning of the study, all subjects had to indicate to be progressively exhausted based on the chronic stress experience. Women and men ranging in age from 18 years to 65 years with a total PSQ_30_ score above 0.50 were included into the study. Exclusion criteria for participation were supplementation or therapy with dietary supplements or drugs that contained l-Trp, vitamins, or minerals within four weeks prior to and for the duration of the study, therapy with antidepressants (monoamine oxidase inhibitors), hypertension (untreated > 150/90 mmHg, treated > 140/85 mmHg), organic fatigue, phenylketonuria, and chronic diarrhea. The trial profile is shown in Figure 1. The study was approved by the Freiburg International Ethics Commission. Informed consent was obtained from each participant prior to the start of the study. In December 2016, this study was retrospectively registered with the U.S. National Institutes of Health Clinical Trials.gov (NCT02997137).

### 2.3. Study Design

This randomized, double-blind, placebo-controlled intervention trial was performed at the University Hospital Bonn between October 2014 and January 2016. A seven-day pre-randomization run-in period (adaptation phase) with placebo-capsules was used to exclude subjects at risk of poor compliance by capsule counting. All participants were instructed to consume one capsule with 200 mL of water two times a day after breakfast and dinner for seven days. The following 12-week intervention phase was planned with a tolerance range of 84 ± 2 days. Inclusion and exclusion criteria were examined before the run-in period and at the beginning of the dietary intervention phase (baseline). Participants were assigned to one of two intervention groups via computer-generated randomization with a block size of four (at baseline, when it was determined that the inclusion criteria had been fulfilled and the exclusion criteria was not violated.) During the dietary intervention phase, participants were required to take the content of a sachet of supplement (9.4 g of powder dissolved in 200 mL of water) daily in the morning. The composition of the verum and placebo products is shown in Table 1. The verum product was specifically designed to decrease chronic exhaustion conditions. Compliance was verified by sachet counting after four, eight, and 12 weeks. The return of all 30 sachets opened and at least four sachets unopened in at least one of the four-week study periods was considered over- and under-consumption, respectively. Participants were instructed to maintain their habitual diet and typical physical activity level throughout the study. Subjects taking regular medication (e.g., thyroid drugs or antihypertensives) were recommended to continue this treatment without changes. The planned sample size of 30 evaluable subjects per group was based on a power of 80% and a level of significance of 5% to detect the efficacy of verum with subsequent expectation. Kocalevent et al. [41] ascertained a total PSQ_30_ score of 0.30 ± 0.15 in the general population. In this study, with the inclusion criterion of a total PSQ_30_ score above 0.50, a mean total PSQ_30_ score of 0.60 ± 0.20 or more was expected at baseline. After 12 weeks of dietary intervention with verum, a broad normalization of mean total PSQ_30_ score, meaning a reduction of at least 0.25 in the verum group, was suspected. With a reduction in total PSQ_30_ score of at least 0.10 in the placebo group, a Cohen’s effect size of 0.75 was assumed. Primary outcome measures were the intergroup comparisons of the pre-post changes of the PSQ_30_ after eight and 12 weeks.

The stress experienced during the past 4 weeks was measured by the German version of the PSQ_30_ [45] at the end of the run-in-phase/baseline and after eight and 12 weeks of the dietary intervention. The inclusion criterion of total PSQ_30_ score above 0.50 was assessed at the beginning of the run-in-phase and at baseline. Participants answered the PNF at baseline and after 12 weeks of dietary intervention. The VAS was completed at baseline and after eight and 12 weeks.

### 2.4. Anthropometric and Clinical Measurements

Anthropometric and clinical measurements were performed in the morning after an overnight fasting period of at least 12 h and under standardized conditions at baseline and after 12 weeks. Body weight and height were determined on a personal floor scale with a height measuring stick (MPE-HM, KERN, Balingen, Germany). Body mass index (BMI, kg/m^2^) was calculated by dividing weight (kg) by height (m) squared. Waist circumference (WC) was measured via flexible tape at the midpoint between the lower margin of the least palpable rib and the top of the iliac crest [46]. Waist-to-height ratio (WHtR) was calculated. After a 10-min resting period in a quiet place and in a relaxed, seated position with the cuff at heart level, three successive measurements of blood pressure (BP) and heart rate were obtained using a digital automatic upper arm BP monitor (model M10-IT, Omron Healthcare Europe B.V., Hoofddorp, The Netherlands) [47]. These accumulated clinical measurements were averaged to determine systolic and diastolic BP and resting heart rate.

### 2.5. Biochemical Analysis

The participants received detailed instruction in written and oral form for gaining saliva-cortisol samples, 24-h-urine collection, accurate recording of dietary intakes, and avoiding serotonin- and/or tryptophan-rich foods 2–3 days before blood sampling due to the determination of serum serotonin.

Laboratory Schottdorf MVZ GmbH (Augsburg, Germany) conducted almost all laboratory analyses. Saliva samples were collected in the evening (between 10 and 11 p.m.) and morning (30 min after awakening) prior to dietary intervention and after 12 weeks. The collections were performed according to the laboratory instructions. Subjects were requested in advance not to eat, drink, or smoke at least 30 min before saliva collection in the evening and in the morning to collect after an overnight fasting period. All saliva samples were provided prior to tooth brushing by using Cortisol-Salivette^®^ (Sarstedt, Nümbrecht, Germany). Salivary cortisol concentrations were determined using Cortisol ELISA (IBL, Hamburg, Germany) and the microplate reader SUNRISE (Tecan, Crailsheim, Germany). Venous blood samples were obtained after an overnight fasting period of at least 12 h at baseline and after 12 weeks. Serum glucose (hexokinase method), glycated hemoglobin (HbA1_c_; turbidimetric immunologic inhibition assay), insulin (ECLIA), gamma-glutamyltransferase (GGT; IFCC method), total cholesterol (TC; CHOD-PAP method), low-density lipoprotein-cholesterol (LDL-C) and high-density lipoprotein-cholesterol (HDL-C; enzymatic color test), triglycerides (TG; GPO-PAP method) and sensitive C-reactive protein (CRP; turbidimetry) were measured using a Roche analyzer. Diagnosis of the metabolic syndrome was performed [48]. Insulin and glucose values were used to calculate the homeostasis model assessment (HOMA-) index, which is a measure of insulin resistance. Serum serotonin was analyzed by HPLC (Merck Hitachi LG6200A) with the electrochemical detector 3000 (Recipe, Munich, Germany). Serum amino acids were determined by HPLC with the Agilent 1100 Series Fluorescence Detector.

The 24-h urinary sodium and chloride excretion were analyzed by an ion sensitive electrode (ISE; Modular, Roche, Basel, Switzerland). Photometry and atomic absorption spectrometry (AAS) were used to detect the 24-h urinary calcium (Modular, Roche) and magnesium concentration (Solaar S4, Thermo Fisher Scientific, Waltham, MA, USA), respectively. Urinary pH value was analyzed at the laboratory of the University Stone Centre, Department of Urology, University of Bonn, Germany.

The self-reported dietary intake (three-day food records) data were reviewed with each participant at baseline and after 12 weeks. These food records were kept in the week prior to baseline and in week 12. PRODI 6.4 basis software (WVG, Stuttgart, Germany) with database BLS 3.02 was used to calculate the nutrient content of foods. The average of three days was assessed.

### 2.6. Statistical Analysis

Statistical analysis was performed on the basis of the intention to treat (ITT) population and by using SPSS^®^ for Windows (version 22.0, IBM, Armonk, New York, NY, USA). Data are presented as mean ± standard deviation (SD) unless otherwise stated. Medians and interquartile ranges of the outcome measures are reported (Tables S1–S9). Statistical comparisons at baseline and between the pre- and post-intervention differences in unpaired variables of both groups were performed using the nonparametric Mann–Whitney U test. The nonparametric Wilcoxon test was used for pre-post intervention changes within groups. Here, the exact tests for small samples were used. Changes in different parameters and their effects on the metabolism were detected via linear regression analysis. Repeated measures (ANOVA) were determined. Differences in classified variables were tested via Fisher’s exact test. All statistical tests were two-sided. A *p* value of < 0.05 was considered statistically significant.

## 3. Results

### 3.1. Participants

A total of 62 women and men were randomized into the verum group (*n* = 31; 23 women and eight men) and the placebo group (*n* = 31; 21 women and 10 men), of whom 59 participants completed all visits and were included into the ITT population (Figure 1). During the 12-week dietary intervention phase three women terminated the study prematurely. One participant in the verum group discontinued the study because of hospitalization after suffering a severe injury at home. One woman in the placebo group discontinued the study for personal reasons. Shortly after randomization, one participant in the verum group was excluded due to abnormal blood values (e.g., liver transaminases and CRP at baseline). No association with the supplementation was found. Daily dietary intervention products were well tolerated by most participants. Three subjects from the verum group indicated gastrointestinal complaints or nausea. Possible correlations with supplementation were assumed. There was no causal relationship to supplements with further reported temporal adverse events, such as urinary tract infection, acne, respiratory tract infection, and obstipation. During the trial, some participants took oral contraceptives (verum group: *n* = 5; placebo group: *n* = 3), thyroid drugs (verum group: *n* = 4; placebo group: *n* = 5), antihypertensive drugs (verum group: *n* = 2; placebo group: *n* = 1), cholesterol lowering medications (placebo group: *n* = 1) and anti-diabetic medications (placebo group: *n* = 1). There was no significant group difference in the use of medication.

Table 2 shows the baseline characteristics of the participants. At baseline, the expected minimum total PSQ_30_ score was clearly exceeded in both groups. These subjective perceived results were confirmed by the disturbed HPA axis activity, as indicated by morning and evening salivary cortisol concentrations that were elevated and borderline increased, respectively (reference range in the morning: 4–10 ng/mL, elevated: >10 ng/mL; reference range in the evening: <1.6 ng/mL; classified borderline range: 1.6–3.2 ng/mL). Four participants with a low morning cortisol concentration (<4 ng/mL) of whom two participants additionally had a low evening cortisol concentration (<2 ng/mL) were detected only in the verum group. For these participants, adrenal impaired function with burnout occurrence could be assumed. At baseline, no significant intergroup differences were found for the PSQ_30_ score, morning and evening salivary cortisol concentrations, serum serotonin, and anthropometric, clinical, and cardiometabolic risk parameters.

Among all participants, 48% were overweight or obese, 46% had abdominal obesity, and 19% had an elevated BP. At baseline, the level of impulsion (determined by the PNF; the higher the value, the lower the level of impulsion) and the difference in salivary cortisol concentrations (morning–evening) were significantly lower in the verum group than in the placebo group.

Protocol violations in the verum group (*n* = 4) and the placebo group (*n* = 7) led to exclusions from the per protocol (PP) population without significant group differences (Figure 1): An overconsumption of dietary supplement was found in four participants (verum group: *n* = 1; placebo group: *n* = 3), and under-consumption was observed for one of the participants in the placebo group. For seven participants (verum group: *n* = 3; placebo group: *n* = 4), the duration of study differed slightly from protocol by an average of 1.9 days due to short-term postponements of the final study visit by these chronically stressed participants. As a direct consequence, underconsumption was additionally presented by one of the participants in the placebo group.

### 3.2. Questionnaires: the PSQ_30_, the PNF, and the VAS

**Figure 2:**
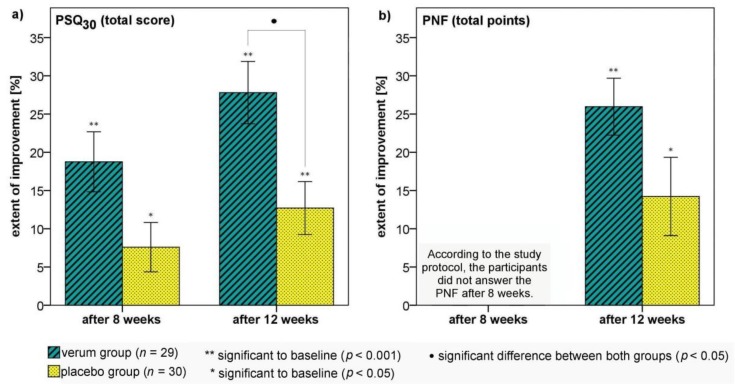
The extent of improvement (%) in perceived stress ((**a**) PSQ_30_) and neurovegetative symptoms ((**b**) PNF) after the eight- and 12-week dietary intervention compared with baseline. Error bars ± 1 SE; •: *U* = 268.5, *p* = 0.012 (Mann-Whitney U test); */**, *p* < 0.05/*p* < 0.001 (Wilcoxon test).

Notably, in the run-in phase, the total PSQ_30_ score remained constant in both groups (data not shown). After the 12-week dietary intervention phase, subjectively perceived stress (PSQ_30_) and neurovegetative symptoms (PNF) improved significantly in the verum and placebo groups (Table 3, Figure 2). Reduced total PSQ_30_ scores were observed in 80% and 88% of all participants after eight (verum group: 90%, placebo group: 70%) and 12 weeks (verum group: 93%, placebo group: 83%), respectively. However, participants in the verum group experienced a significantly lower total PSQ_30_ score than participants of the placebo group after 12 weeks (see the assumed Cohen’s effect size). The influence of baseline and repeated measurements after eight and 12 weeks, respectively, showed no change in the statistical evaluation of the group effects (univariate analysis: *F* = 8.684, baseline-adjusted: *F* = 8.561, repeated measurements: *F* = 5.771; *p* < 0.05, respectively). At the end of the trial, the mean total PSQ_30_ score was below the inclusion criterion of > 0.50 only in the verum group. After eight weeks, no significant group difference was observed for change in total PSQ_30_ score, but a significant group difference was found in the overload scale (*U* = 305.5, *p* = 0.046). The improvements in perceived overload after eight and 12 weeks and of perceived irritability, lack of joy, fatigue and tension after 12 weeks were significantly greater in the verum group than in the placebo group. Moreover, participants in the verum group achieved the initially suspected reduction in total PSQ_30_ score of at least 0.25 at significantly higher rates than participants of the placebo group after eight (verum group: *n* = 5, placebo group: *n* = 0; *p* = 0.024) and 12 weeks (verum group: *n* = 12, placebo group: *n* = 2; *p* = 0.002).

After the 12-week dietary intervention phase, the total PNF points improved significantly in both the verum and placebo groups without group differences (Table 3, Figure 2). The categories psycho-neurovegetative stability and neurological symptoms significantly improved in both groups. The categories impulsion, excitability, and concentration were significantly positively affected only in the verum group after 12 weeks compared with baseline. The change in ranking for the impulsion category was significantly better in the verum group than in the placebo group.

After 12 weeks, there was no significant intergroup difference in the pre- to post-total VAS points (Table 3). The results of the PSQ_30_, PNF, and VAS correlated significantly in both groups (Table S10).

### 3.3. Salivary Cortisol, Serum Amino Acid, and Serotonin Concentrations

**Figure 3:**
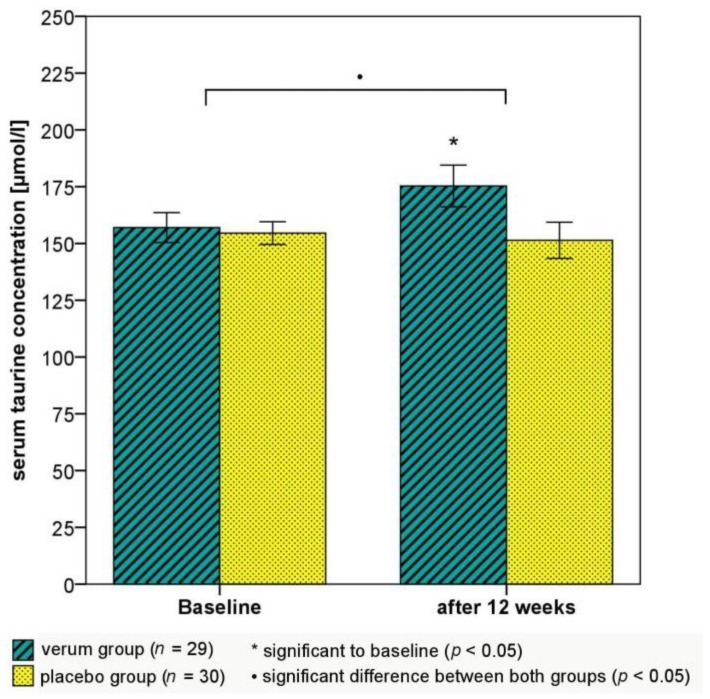
Effects of the verum and placebo interventions on serum taurine concentration. Error bars ±1 SE; •: *U* = 290.0, *p* = 0.027 (Mann–Whitney U test); * *p* = 0.016 (Wilcoxon test).

Salivary cortisol concentration in the morning and evening were not significantly changed in the verum and placebo groups (Table 4). After 12 weeks, four participants in the verum group with a low morning cortisol concentration (<4 ng/mL) and two of them with initial suspected burnout syndrome (see Section 3.1. for more details) had no longer low morning and evening (<2 ng/mL) salivary cortisol concentrations. In the placebo group, no low cortisol concentrations were observed during the study. However, no significant intergroup difference was detected. Furthermore, participants in the verum group showed a favorable significant increase in quotients of serum l-Trp with CAA and BCAA. In the verum and placebo groups, blood analysis showed no serum serotonin deficiency. The serum serotonin concentration did not change during the verum treatment, whereas a significant reduction was observed during the placebo treatment (significant intergroup difference: *U* = 304.0, *p* = 0.047; Table 4). In the placebo group, the serum magnesium concentration decreased to a significantly higher extent than in the verum group with a constant mean magnesium concentration (verum group: −0.00 ± 0.04 mmol/L (*p* = 0.604), placebo group: −0.03 ± 0.05 mmol/L (*p* = 0.004); *U* = 283.0, *p* = 0.021). Moreover, after the verum treatment, participants showed a significantly higher taurine concentration (Figure 3), serum folic acid concentration, and 24-h urinary magnesium excretion than at baseline, whereas the placebo treatment did not affect these parameters (Table 4). Significant intergroup differences were observed for all these changes. In the entire ITT population, changes in the serum serotonin concentration were positively correlated with changes in the serum magnesium concentration (RCB = 217.6, *p* = 0.005) and the serum taurine concentration (RCB = 0.211, *p* = 0.019) during the 12-week intervention phase (age-adjusted).

### 3.4. Anthropometric, Clinical, Cardiometabolic, and Biochemical Parameters

The verum and placebo treatments significantly reduced systolic BP from baseline to the end of the intervention (Table 5). The change in resting heart rate for men in the verum group was significantly better than that for men in the placebo group (*U* = 11.5, *p* = 0.011). These results demonstrate positive effects on HPA axis activity in men. In the placebo group, body weight, BMI, WC, and WHtR increased significantly during 12 weeks, whereas participants in the verum group were unaffected (Table 5). No significant inter-group difference was observed.

Neither verum nor placebo consumption resulted in significant changes in lipid and carbohydrate metabolism (Table 5). In both groups, no significant changes were observed in the number of participants with elevated fasting plasma glucose and metabolic syndrome after 12 weeks. However, in the placebo group, the number of participants with metabolic syndrome and elevated fasting plasma glucose nearly doubled after 12 weeks. This rate of change did not represent the typical course. In the placebo group, weight gain and the change in the folic acid concentration were significantly correlated with the change in the HOMA-index (Table S10). The concentrations of ferritin and ALAT decreased to a significantly higher extent in the verum group than in the placebo group without clinical relevance in mean results.

No significant 12-week change in dietary intake of energy, protein, amino acids, fat, and carbohydrates was observed in both groups, without significant intergroup differences (Table S11). However, after taking the verum supplement over a 12-week period, the dietary intake of fiber, vitamin C, and potassium significantly increased, whereas the dietary intake of cholesterol significantly decreased only in the verum group. Thus, significant differences at the changes in the dietary intake of fiber, vitamin C, potassium, and cholesterol were observed between both groups.

## 4. Discussion

The study showed that the daily supplementation of the specific amino acid composition with vitamins and minerals resulted in a significantly greater improvement in subjective perceived stress as measured by the total PSQ_30_ score compared with the placebo group after the 12-week intervention. The findings from this study were consistent with results from previous studies. A meta-analysis has documented beneficial effects of supplementation with micronutrients on perceived stress and mental fatigue in apparently healthy individuals [40]. Vitamins and minerals play an important role in brain health; they act as cofactors in the synthesis and metabolism of neurotransmitters, which regulate the neuronal systems. In particular, formulas containing many B vitamins [49] in relatively high doses [40], such as the present verum supplement, showed the impact of B vitamins in managing stress. One explanation for the effectiveness of micronutrient supplements might be an inadequate nutritional intake due to prolonged stress [35]. The effects of specific amino acid supplements in nutrition therapy on mental health are of important interest and are increasingly examined in intervention studies [32,50].

The PSQ in original and reduced size is one of the most frequently used instruments for assessing subjectively perceived stress in men and women [51,52]. Previous studies have used the PSQ to measure chronic stress perception [53,54]. In a representative sample of the German general population with a mean total PSQ_30_ score of 0.30 (SD 0.15), the prevalence rate was estimated to be 14.5% for an elevated stress experience (total PSQ_30_ score > 0.45 – ≤ 0.59) and 3.1% for a high stress experience (total PSQ_30_ score ≥ 0.60) [41,55]. When comparing these data with the mean total PSQ_30_ score of 0.669 ± 0.119 in the present ITT population at baseline, this study included a specially selected group of participants with high subjective perceived stress as expected. The longitudinal data from Lindgren et al. [56] revealed that individuals with long-term stability in the total PSQ_30_ score above 0.34 (the moderate and high stress group) had significantly smaller hippocampal volumes, which may be a vulnerability factor for stress-related disorders, compared with those in the low perceived stress group. Kato et al. [57] suggested that both stress and emotional instability are plausible mechanisms for chronic fatiguing illness, whereas emotional instability has endogenous, moderating effects mediated by familial factors and stress has exogenous, direct effects. Moreover, higher self-reported stress is associated with higher risk of chronic fatigue-like illness (odds ratios, 1.64–5.81) [57], burnout, anxiety, depression and resilience [58]. However, the verum group in our study showed significantly greater reductions in the PSQ_30_ subscales of overload, irritability, lack of joy, fatigue, and tension than the placebo group. After 12 weeks, 25% more participants from the verum group compared with the placebo group achieved total PSQ_30_ scores below the corresponding inclusion criterion (the total PSQ_30_ score > 0.50). Thus, the analysis revealed that significantly more participants in the verum group reported a reduction of at least 0.25 of the total PSQ_30_ score compared with participants in the placebo group after 8 and 12 weeks. The PSQ is also associated with neurovegetative complaints [52]. This was demonstrated by the fact that in both groups, the changes in total PSQ_30_ scores were correlated significantly and positively with the changes in the total points of the PNF and VAS after 12 weeks. However, group differences were not yet observed at these parameters during the 12-week dietary intervention, but the subscales impulsion (PNF) and productivity (VAS) were more favorably affected in the verum group compared with the placebo group. Thus, the present results suggest that the intake of the specific amino acid composition with micronutrients decreased the perception of chronic stress in a complex way.

The HPA axis is a vital part of the human stress response system. Therefore, cortisol regulation is a key topic in psychobiological stress research [59]. In the present study, the initially reported high chronic stress levels corresponded with the elevated salivary cortisol results in the morning in both groups. Thus, a hyperactive HPA axis function was detected at baseline. However, under chronic stress this system is at high risk of turning to hypoactive functioning when a state of exhaustion is reached and the individual can no longer cope with stress [60]. Two participants in the verum group already exhibited such a hypoactive HPA axis function at baseline that is comparable with those of most burnout patients [60,61,62]. After 12 weeks, HPA axis activity recovered for both participants. However, despite significant reductions in perceived stress, the mean salivary cortisol concentrations in the morning and evening were not significantly affected by the dietary interventions. Thus, no significant effect on this HPA axis marker [18,63] was found after the study. Over the last decades, salivary cortisol has been regarded as a useful and valid biomarker in stress research, which represents the reliable reflection of the respective unbound cortisol in blood [64]. However, numerous factors can influence cortisol response [59]. Van Holland et al. [65] concluded that physiological stress effects assessed from saliva cannot be used interchangeably with self-reported stress in a working population, because they correlate only weakly. Recently, the use of hair cortisol as a potential biomarker of chronic stress appeared to be better for evaluating the effectiveness of a stress reduction program compared with salivary cortisol [66,67,68].

Serotonin is one of the most powerful neurotransmitters, with widespread effects in behavioural and neuropsychological processes including mood, cognition, perception, stress responses, appetite, memory, and sleep/circadian rhythms. In the periphery, serotonin acts as a gastrointestinal regulating agent and a modulator of vasoconstriction/dilation [69]. The main determinant of central serotonin synthesis is the ratio of free l-Trp to the sum of CAA. This quotient can be increased by l-Trp treatment [31,70]; consuming a carbohydrate-rich and protein-poor meal causes insulin to be secreted or by physical activity [71,72,73]. Insulin secretion and physical activity result in an uptake of CAA in muscles, followed by a decrease in these plasma concentrations. Moreover, the increase in plasma free fatty acids results in a higher free l-Trp concentration because binding to albumin is inhibited [74,75,76]. The l-Trp/BCAA-ratio and l-Trp/CAA-ratio significantly increased after dietary intervention in the verum group, although the present verum supplement contained the two CAA l-tyrosine and l-phenylalanine. Thus, a stimulation of central serotonin synthesis could be assumed. However, the reason for this increase is uncertain. The verum supplement did not contain l-Trp and, as expected, no change was detected in the serum l-Trp concentration. However, a 40–70% increase in the plasma l-Trp/CAA-ratio of chronically stressed subjects may be required to stimulate central serotonin synthesis [77,78]. The participants in the verum group of the present study did not achieve that assumed requirement.

The relationship between peripheral and central serotonergic systems has been discussed in mice and humans [79]. Some studies documented no association between the serotonin concentrations in and outside the central nervous system [31]. However, plasma and cerebrospinal fluid concentrations of serotonin seem to be closely related in stroke patients with or without depression [80]. In the present study, the plasma serotonin concentration significantly decreased to a higher extent in the placebo group than in the verum group after 12 weeks. A positive correlation with the significantly reduced plasma magnesium concentration was observed in this group (RCB = 262.3, *p* = 0.025). Thus, in comparison with the results in the verum group, the verum supplement seemed to have preventive effects on plasma magnesium homeostasis and serotonin synthesis in participants with chronic stress. However, the specific combination of amino acids, vitamins and minerals, including magnesium and several other important cofactors of the serotonin synthesis [35], is supposed to have synergistic effects in this connection, because no significant association between the plasma concentrations of serotonin and magnesium was observed in the verum group.

Serotonin metabolism is also controlled by taurine, glycine, GABA, and glutamate in vitro [81]. A significant association between changes in plasma serotonin and taurine was observed only in the placebo group (RCB = 0.332, *p* = 0.024). Plasma taurine concentration increased significantly in the verum group and did not change in the placebo group. Thus, an effect of taurine on central serotonin synthesis and enhanced stress tolerance were supposed in the verum group. Potential beneficial actions of taurine in hypertension, atherosclerosis, and diabetic cardiomyopathy have been discussed [82,83]. No correlation between changes in plasma taurine and systolic BP was observed in the present study. However, a previous study revealed that plasma amino acids do not necessarily reflect changes in amino acid turnover, therefore they should be interpreted with caution [84]. Thus, these results showed that the present verum supplement and the impact on lifestyle factors decreased subjectively perceived chronic stress and these laboratory parameters in a complex way.

Brain serotonin dysfunction is not only associated with psycho-neurovegetative symptoms, mood disorders, and impaired stress coping but also with decreased control of overall energy intake [70]. High levels of stress in individuals are associated with increased consumption of comfort food. Thus, greater perceived stress was associated with lower fruit, vegetable, and protein intake, greater consumption of salty snacks and lower participation in physical activities [85,86]. Repeated stimulation of the reward pathways through stress-induced HPA axis stimulation, intake of highly palatable food, or both may lead to neurobiological adaptations that promote the compulsive nature of overeating. Cortisol may influence the reward value of food via neuroendocrine/peptide mediators such as leptin, insulin, and neuropeptide Y. By contrast, glucocorticoids are acutely antagonized by insulin and leptin. Under chronic stress, this finely balanced system is dysregulated, possibly contributing to increased food intake and visceral fat accumulation [24,87,88]. In the present study, this effect was detected only in the placebo group with significant increases in WC, WHtR, body weight, and BMI after 12 weeks. Reciprocally, obesity promotes a systemic low-grade inflammation state, which can chronically stimulate and disturb the stress system. This vicious cycle probably initiates visceral adipose tissue dysfunction, which might be the trigger for the development of metabolic syndrome [17,19,20,89]. The health risks associated with chronic psychological stress are cardiovascular disease, atherosclerosis [90], diabetes mellitus type 2 [20], adverse immune effects [91], and increased risk of mortality [92]. Thus, the present results indicated that participants in the verum group were prevented from an increased stress-related eating behavior with associated effects on anthropometric and cardiometric parameters. Additional beneficial effects caused by the optimized dietary intake of fruits, vegetables and animal-based foods, without effects on the macronutrients, are assumed. Whether this optimized food intake is causally related to the verum treatment or these participants underwent lifestyle changes outside of study instructions is questionable. In both groups, a significant reduction in systolic BP, a SAM axis marker [63], was observed. Therefore, a positive impact on the SAM axis activity is assumed. After 12 weeks, the resting heart rate increased above the cutoff of 70 beats/min [93] only in men of the present placebo group. An elevated resting heart rate is an independent predictor of coronary artery disease and stroke [93]. The opposing courses in resting heart rate in men of both groups indicate an increased cardiovascular risk in the placebo group. However, the dietary interventions did not significantly affect the mean values of the lipid and glucose metabolism in both groups. However, in the placebo group, the almost redoubling of the number of participants with the metabolic syndrome and elevated fasting plasma glucose concentrations are noteworthy and show the risk of chronic stress for metabolic dysfunction in accordance with other studies [94,95]. At the beginning and end of this study, inflammatory processes were elevated in both groups. Human intervention studies with synergistic actions of nutrients and whole diets, such as the Mediterranean-style diet, are needed to investigate the benefits for mental and cardiometabolic health [96].

During the 12-week dietary intervention phase, the significant change in plasma ferritin concentration in the verum group could not be explained by gender segregation or age groups (data not shown). The verum supplement did not contain iron but zinc. In previous studies, an inhibitory effect of oral zinc supplementation on iron absorption was supposed, possibly due to the antagonistic action of zinc during iron absorption from the gastrointestinal tract [97,98,99]. Iron absorption might also be reduced by the increased intake of plant-based foods including their inhibitors of iron absorption and dietary iron form with a lower bioavailability than in meat [100].

The small number of cases is a limiting factor. The use of the PSQ_30_ for participant selection and assessment of the primary outcome was necessary, because of the small sample size of the present pilot study and the fact that a large number of study participants without an improvement potential would obliterate the therapeutic effect. However, this procedure can introduce bias. While other measures, such as salivary cortisol, provide a complimentary and adequate approach for examining stress, the manner in which cortisol was measured and the reliability of subject-collected saliva limited its utility in this study. Therefore, saliva collection should be performed under more controlled conditions. Besides plasma folic acid and magnesium concentrations, no other biochemical blood measures of vitamins and minerals were measured. For example, the concentrations of B vitamins and zinc, as well as their relation to changes in perceived stress, plasma serotonin, cardiometabolic risk factors, and plasma ferritin, would be of great interest. These would clarify the interpretation of the extent to which the verum supplement was related to primary and secondary outcomes. The change in plasma ferritin should be examined in other studies with comparable supplements. During further studies, changes in physical activity and personal situation should be assessed in addition to the nutrition status. More studies with a larger cohort are necessary to determine further associations and effects.

## 5. Conclusions

Daily supplementation with a specific amino acid composition with micronutrients in participants with chronic perceived stress and exhaustion conditions resulted in more beneficial effects compared with the placebo group after 12 weeks. In the verum group, the reported subjectively perceived stress and the PSQ_30_ subscales of overload, irritability, lack of joy, fatigue, and tension were reduced to a greater extent than those in the placebo group. Improvements in neurovegetative symptoms were similar in both groups. However, the HPA axis was not significantly affected. In the verum group, favorable effects on the serotonergic system were assumed by the increase in the l-Trp/CAA ratio, the l-Trp/BCAA ratio, and several verum ingredients that act synergistically as cofactors in serotonin synthesis. In addition, the verum treatment had preventive effects on serum magnesium homeostasis, which was disturbed in the placebo group due to decreased serum concentration. Cardiometabolic risk factors, such as body weight, BMI, WC, and WHtR, increased significantly in the placebo group and resulted in a doubling of subjects with metabolic syndrome after 12 weeks compared with baseline. The cardiometabolic risk of participants in the verum group did not significantly deteriorate. Additional positive effects of the optimized food intake of fruits, vegetables, and animal-based foods causally related to the verum treatment or lifestyle changes were supposed. Further research about the complex interaction between specific nutrients and other lifestyle factors, such as food choice, would be of interest.

## Figures and Tables

**Table 1:** Composition of dietary intervention products per day (9.4 g powder).

	Verum	Placebo
Energy	114 kJ/27 kcal	15 kJ/3 kcal
Protein	4.2 g	>0.0 g
Carbohydrates (sugar)	0.529 g (0.0 g)	0.344 g (0.0 g)
Fat	0.0 g	>0.0 g
Dietary fibers	0.002 g	0.001 g
**Amino acids**		
Taurine	1000 mg	-
l-ornithine	2000 mg	-
l-phenylalanine	200 mg	-
l-tyrosine	1000 mg	-
**Vitamins**		
Vitamin C	300 mg	-
Vitamin B_1_	25 mg	-
Vitamin B_2_	25 mg	-
Vitamin B_6_	25 mg	-
Vitamin B_12_	50 µg	-
Niacin	100 mg	-
Pantothenic acid	100 mg	-
Folic acid	800 µg	-
ß-carotene ^1^	0.8 mg	1 mg
**Minerals**		
Magnesium	300 mg	-
Zinc	15 mg	-
Selenium	100 µg	-
Chrome	50 µg	-
Molybdenum	50 µg	-
Further ingredients	citric acid; maltodextrin; aroma; acesulfame K; silicic acid	erythritol; citric acid; maltodextrin; aroma; acesulfame K; silicic acid

Abbreviations: ^1^ coloring agent; verum supplement: a dietary food for special medical purposes (Article 2(2) (g) of the Regulation (EU) No. 609/2013).

**Table 2:** Baseline characteristics of participants with chronic psychological stress.

	Verum Group (*n* = 29) ^1^	Placebo Group (*n* = 30) ^1^	V vs. P
	Mean ± SD; *n* (%)	Mean ± SD; *n* (%)	*p*-Value
Women	21 (72.4%)	20 (66.7%)	0.779
Men	8 (27.6%)	10 (33.3%)	
Age (years)	43.6 ± 12.6	45.0 ± 11.9	0.693
Smokers	7 (24.1%)	8 (26.7%)	1.000
**Anthropometric, clinical, and cardiometabolic parameters**			
Height (cm)	170.5 ± 8.7	170.4 ± 9.6	0.795
Weight (kg)	73.3 ± 13.3	76.2 ± 21.4	0.913
BMI (kg/m^2^)	25.2 ± 4.4	25.9 ± 5.8	0.982
BMI 25–< 30 kg/m²	10 (34.5%)	7 (23.3%)	0.399
BMI ≥ 30 kg/m²	4 (13.8%)	7 (23.3%)	0.506
WC (cm; women)	87.9 ± 13.5	85.2 ± 11.7	0.473
WC ≥ 88 cm (women)	11 (52.4%)	8 (40%)	0.536
WC (cm; men)	96.4 ± 6.6	106.0 ± 16.4	0.051
WC ≥ 102 cm (men)	2 (25%)	6 (60%)	0.188
BP systolic (mmHg)	113.7 ± 12.6	111.2 ± 15.8	0.352
BP diastolic (mmHg)	75.1 ± 8.2	75.7 ± 9.0	0.704
BP ≥ 130/85 mmHg	6 (20.7%)	5 (16.7%)	0.748
Resting heart rate (1/min)	66.7 ± 9.0	66.5 ± 8.4	0.877
TC (mg/dL)	209.9 ± 51.7	213.1 ± 43.2	0.606
HDL-C (mg/dL)	64.3 ± 18.6	64.4 ± 21.7	0.789
LDL-C (mg/dL)	130.1 ± 48.0	137.2 ± 35.1	0.356
TG (mg/dL)	109.2 ± 64.9	92.7 ± 41.8	0.427
FPG (mg/dL)	88.1 ± 9.3	91.4 ± 22.6	0.979
HbA1_c_ (%)	5.41 ± 0.29	5.63 ± 0.75	0.380
HOMA-index	1.50 ± 0.84	1.96 ± 2.04	0.574
Insulin-ECLIA (μU/mL)	6.75 ± 3.54	7.83 ± 4.90	0.496
CRP sensitive (mg/L)	2.86 ± 3.89	1.65 ± 2.97	0.061
**Serum serotonin and salivary cortisol**			
Serum serotonin (μg/L)	137.7 ± 58.0	150.1 ± 80.9	0.554
Cortisol_morning (ng/mL)	12.54 ± 6.61	14.62 ± 4.55	0.118
Cortisol > 10 ng/mL (m)	20 (69.0%)	25 (83.3%)	0.233
Cortisol_evening (ng/mL)	3.00 ± 4.90	2.04 ± 2.49	0.313
Cortisol > 2 ng/mL (e)	11 (37.9%)	7 (23.3%)	0.267
Δ cortisol (m − e) (ng/mL)	9.54 ± 6.37	12.57 ± 5.35	0.037
**PSQ_30_ and PNF**			
Total PSQ_30_ score	0.674 ± 0.124	0.664 ± 0.116	0.836
Total PNF (points)	53.7 ± 13.2	48.3 ± 10.4	0.104
Impulsion (points)	11.8 ± 3.7	9.8 ± 3.3	0.016
**Serum amino acids**			
l-ornithine (µmol/L)	106.7 ± 33.0	100.3 ± 21.3	0.766
l-phenylalanine (µmol/L)	95.6 ± 19.8	99.6 ± 13.2	0.467
Taurine (µmol/L)	157.0 ± 35.5	154.6 ± 27.4	0.913
l-Trp (µmol/L)	53.0 ± 14.8	54.9 ± 9.4	0.213
l-tyrosine (µmol/L)	67.5 ± 17.4	70.4 ± 13.1	0.375
l-Trp/CAA	0.087 ± 0.033	0.086 ± 0.013	0.456
l-Trp/BCAA	0.117 ± 0.040	0.118 ± 0.021	0.429

Abbreviations: BCAA, branched-chain amino acids (the sum of l-valine, l-leucine, and l-isoleucine); BMI, body mass index; BP, blood pressure; Δ cortisol (m − e); cortisol difference morning–evening; CAA, competing amino acids (the sum of the BCAA, l-phenylalanine, and l-tyrosine); CRP, C-reactive protein; ECLIA, electrochemiluminescence immunoassay; FPG, fasting plasma glucose; HbA1_c_; glycated hemoglobin A1_c_; HDL-C, high-density lipoprotein cholesterol; HOMA-index, homeostasis model assessment index; LDL-C, low-density lipoprotein cholesterol; l-Trp; l-tryptophan; PNF, Psychological Neurological Questionnaire; PSQ, Perceived Stress Questionnaire; SD, standard deviation; TC, total cholesterol; TG, triglycerides; WC, waist circumference; ^1^ Intention-to-treat (ITT) population; *p*-Value: Mann–Whitney U test or Fisher’s exact test.

**Table 3:** Values of the PSQ_30_, PNF, and VAS before and during the dietary intervention.

	Verum Group (*n* = 29) ^1^					Placebo Group (*n* = 30) ^1^					V vs. P	
	Mean ± SD					Mean ± SD					*p*-Value ^b^	
	Baseline	Week 8	Week 12	Difference 8 Weeks	Difference 12 Weeks	Baseline	Week 8	Week 12	Difference 8 Weeks	Difference 12 Weeks	8 Weeks	12 Weeks
**Total PSQ_30_ score**	0.674 ± 0.124	0.543 ± 0.160	0.482 ± 0.163	−0.131 ± 0.143 **	−0.192 ± 0.161 **	0.664 ± 0.116	0.607 ± 0.127	0.581 ± 0.163	−0.057 ± 0.115 *	−0.083 ± 0.121 **	0.092	0.012
Harassment	2.8 ± 0.5	2.4 ± 0.5	2.2 ± 0.6	−0.4 ± 0.6 *	−0.6 ± 0.7 **	2.8 ± 0.5	2.6 ± 0.6	2.5 ± 0.6	−0.2 ± 0.6	−0.3 ± 0.6 *	0.370	0.067
Overload	3.1 ± 0.5	2.7 ± 0.6	2.6 ± 0.7	−0.4 ± 0.4 **	−0.5 ± 0.5 **	3.1 ± 0.5	3.0 ± 0.5	2.9 ± 0.7	−0.1 ± 0.5	−0.2 ± 0.5 *	0.046	0.014
Irritability	3.0 ± 0.6	2.7 ± 0.7	2.4 ± 0.6	−0.3 ± 0.7 *	−0.6 ± 0.7 **	2.8 ± 0.6	2.8 ± 0.6	2.6 ± 0.6	−0.0 ± 0.6	−0.2 ± 0.6 *	0.143	0.030
Lack of joy	3.0 ± 0.4	2.7 ± 0.5	2.6 ± 0.4	−0.3 ± 0.5 *	−0.4 ± 0.4 **	3.0 ± 0.5	2.9 ± 0.4	2.9 ± 0.6	−0.1 ± 0.3	−0.1 ± 0.4 *	0.199	0.006
Fatigue	3.3 ± 0.4	2.9 ± 0.6	2.7 ± 0.5	−0.4 ± 0.7 *	−0.6 ± 0.6 **	3.3 ± 0.4	3.0 ± 0.5	3.0 ± 0.5	−0.2 ± 0.4*	−0.3 ± 0.5 *	0.339	0.034
Worries	2.8 ± 0.6	2.3 ± 0.7	2.2 ± 0.6	−0.5 ± 0.5 **	−0.6 ± 0.6 **	2.8 ± 0.4	2.6 ± 0.4	2.5 ± 0.6	−0.2 ± 0.4*	−0.4 ± 0.5 **	0.121	0.140
Tension	3.1 ± 0.4	2.6 ± 0.6	2.4 ± 0.7	−0.5 ± 0.5 **	−0.7 ± 0.6 **	3.0 ± 0.5	2.8 ± 0.6	2.7 ± 0.6	−0.2 ± 0.6	−0.3 ± 0.6 *	0.077	0.012
**Total PNF points**	53.7 ± 13.2	^a^	39.3 ± 14.4	-	−14.3 ± 11.8 **	48.3 ± 10.4	^a^	41.3 ± 14.6	-	−7.0 ± 12.5 *	-	0.063
Psycho-neurovegetative stability	19.0 ± 5.2	^a^	13.3 ± 4.7	-	−5.7 ± 4.7 **	17.9 ± 4.4	^a^	14.6 ± 5.2	-	−3.3 ± 5.2 *	-	0.136
Neurological symptoms	7.2 ± 3.7	^a^	4.6 ± 2.9	-	−2.6 ± 2.5 **	6.3 ± 3.5	^a^	4.8 ± 3.4	-	−1.5 ± 3.2 *	-	0.246
Impulsion	11.8 ± 3.7	^a^	8.4 ± 4.5	-	−3.4 ± 3.6 **	9.8 ± 3.3	^a^	9.1 ± 4.1	-	−0.7 ± 3.4	-	0.010
Excitability	5.7 ± 2.9	^a^	4.5 ± 2.9	-	−1.2 ± 2.2 *	4.9 ± 2.0	^a^	4.3 ± 2.4	-	−0.6 ± 2.0	-	0.334
Concentration and memory	9.9 ± 3.7	^a^	8.5 ± 3.9	-	−1.4 ± 3.0 *	9.4 ± 2.9	^a^	8.5 ± 3.2	-	−0.9 ± 2.9	-	0.507
**Total VAS points**	15.9 ± 3.6	14.1 ± 3.7	12.7 ± 4.4	−1.8 ± 3.9	−3.3 ± 4.2 **	15.3 ± 3.6	15.0 ± 3.8	14.5 ± 4.3	−0.3 ± 4.6	−0.8 ± 5.5	0.284	0.178
Lack of motivation	3.1 ± 1.0	2.7 ± 0.8	2.4 ± 1.1	−0.4 ± 1.1	−0.7 ± 1.1 *	3.2 ± 0.9	2.9 ± 1.0	2.9 ± 1.0	−0.3 ± 1.2	−0.4 ± 1.3	0.671	0.558
Indifference	3.0 ± 1.0	2.4 ± 1.1	2.2 ± 1.0	−0.5 ± 1.3 *	−0.8 ± 1.1 *	2.8 ± 1.0	2.8 ± 1.0	2.5 ± 1.1	−0.0 ± 1.2	−0.3 ± 1.5	0.155	0.235
Fatigue	3.4 ± 1.1	3.1 ± 1.1	2.8 ± 1.2	−0.3 ± 1.3	−0.6 ± 1.3 *	3.5 ± 1.0	3.4 ± 1.1	3.5 ± 1.0	−0.1 ± 1.4	0.0 ± 1.3	0.728	0.096
Mood changes	3.2 ± 0.9	2.9 ± 1.0	2.7 ± 1.0	−0.3 ± 1.1	−0.5 ± 1.1 *	2.8 ± 1.0	2.9 ± 1.1	2.6 ± 1.1	0.1 ± 1.2	−0.2 ± 1.5	0.250	0.389
Productivity	3.1 ± 1.1	2.9 ± 0.8	2.5 ± 1.0	−0.2 ± 0.9	−0.6 ± 1.1 *	2.9 ± 0.9	3.0 ± 1.0	3.0 ± 0.9	0.1 ± 1.2	0.0 ± 1.2	0.364	0.040

Abbreviations: PSQ, Perceived Stress Questionnaire; PNF, Psychological Neurological Questionnaire; VAS, Visual Analogue Scales; SD, standard deviation; ^1^ Intention to treat (ITT) population; ^a^ not implemented according to the study protocol; *p*-Value: **, *p* < 0.001; *, *p* < 0.05 (Wilcoxon test within groups), ^b^ Mann–Whitney U test.

**Table 4:** Changes in salivary cortisol, serum amino acid, serotonin, mineral, and vitamin concentrations during the dietary intervention.

	Verum Group (*n* = 29) ^1^			Placebo Group (*n* = 30) ^1^			V vs. P
	Mean ± SD			Mean ± SD			*p*-Value ^a^
	Baseline	Week 12	Difference	Baseline	Week 12	Difference	12 Weeks
**Salivary**							
Cortisol_morning (ng/mL)	12.54 ± 6.61	13.96 ± 5.76	1.42 ± 8.17	14.62 ± 4.55	15.08 ± 6.64	0.47 ± 5.78	0.596
Cortisol_evening (ng/mL)	3.00 ± 4.90	2.07 ± 1.80	−0.93 ± 5.0	2.04 ± 2.49	2.59 ± 3.60	0.55 ± 4.10	0.495
Δ cortisol (m − e) (ng/mL)	9.54 ± 6.37	11.89 ± 6.05	2.34 ± 7.32	12.57 ± 5.35	12.49 ± 7.98	−0.08 ± 7.60	0.300
**Serum**							
Protein (g/dL) †	7.15 ± 0.34	7.16 ± 0.40	0.01 ± 0.36	7.41 ± 0.43	7.24 ± 0.44	−0.17 ± 0.38 *	0.075
l-arginine (µmol/L) †	115.9 ± 27.9	122.7 ± 25.4	6.8 ± 19.4	132.7 ± 22.8	126.3 ± 19.5	−6.4 ± 23.1	0.014
l-ornithine (µmol/L)	106.7 ± 33.0	108.4 ± 67.4	1.8 ± 52.0	100.3 ± 21.3	100.4 ± 32.5	0.1 ± 22.5	0.590
l-phenylalanine (µmol/L)	95.6 ± 19.8	97.5 ± 14.5	1.9 ± 17.9	99.6 ± 13.2	94.9 ± 15.9	−4.6 ± 15.7	0.090
Taurine (µmol/L)	157.0 ± 35.5	175.3 ± 49.5	18.3 ± 38.2 *	154.6 ± 27.4	151.4 ± 43.6	−3.2 ± 40.1	0.027
l-Trp (µmol/L)	53.0 ± 14.8	53.1 ± 9.3	0.1 ± 14.9	54.9 ± 9.4	54.6 ± 9.8	−0.3 ± 7.8	0.197
l-tyrosine (µmol/L)	67.5 ± 17.4	71.4 ± 21.9	3.9 ± 18.6	70.4 ± 13.1	69.2 ± 14.7	−1.2 ± 19.3	0.102
l-Trp/CAA	0.087 ± 0.033	0.088 ± 0.015	0.001 ± 0.033 *	0.086 ± 0.013	0.087 ± 0.015	0.001 ± 0.014	0.181
l-Trp/BCAA	0.117 ± 0.040	0.122 ± 0.021	0.005 ± 0.039 *	0.118 ± 0.021	0.118 ± 0.022	0.000 ± 0.021	0.088
Serotonin (μg/L)	137.7 ± 58.0	138.7 ± 57.3	1.0 ± 20.9	150.1 ± 80.9	140.5 ± 99.8	−9.6 ± 32.4 *	0.047
Folic acid (ng/mL) ^b^	9.67 ± 4.13	18.18 ± 2.84	8.51 ± 4.38 **	9.48 ± 3.08	8.93 ± 3.71	−0.55 ± 2.48	< 0.001
Magnesium (mmol/L)	0.87 ± 0.06	0.87 ± 0.05	−0.00 ± 0.04	0.89 ± 0.06	0.86 ± 0.06	−0.03 ± 0.05 *	0.021
Calcium (mmol/L)	2.38 ± 0.08	2.37 ± 0.09	−0.01 ± 0.10	2.40 ± 0.12	2.37 ± 0.10	−0.03 ± 0.10 *	0.413
**Urine**							
Magnesium (mmol/24 h)	3.83 ± 1.41	5.67 ± 1.49	1.84 ± 1.56 **	3.63 ± 1.27	3.84 ± 1.61	0.21 ± 1.22	< 0.001
Calcium (mmol/24 h)	4.88 ± 2.36	5.60 ± 3.19	0.71 ± 2.58 *	4.58 ± 3.26	4.32 ± 2.93	−0.26 ± 1.56	0.023
Sodium (mmol/24 h)	166.9 ± 66.3	142 ± 58.1	−24.9 ± 65.9 *	157.4 ± 75.7	172.0 ± 84.5	14.7 ± 82.9	0.025
Chloride (mmol/24 h)	151.9 ± 60.8	140.6 ± 56.1	−11.3 ± 59.1	134.3 ± 66.4	148.5 ± 76.6	14.2 ± 70.3	0.145
pH value	6.22 ± 0.42	6.06 ± 0.43	−0.16 ± 0.48	6.16 ± 0.35	6.26 ± 0.42	0.10 ± 0.44	0.028
Creatinine (g/24h)	1.46 ± 0.53	1.37 ± 0.44	−0.09 ± 0.34	1.37 ± 0.52	1.33 ± 0.53	−0.04 ± 0.30	0.949
Volume (L/24h)	2.14 ± 0.69	2.12 ± 0.73	−0.02 ± 0.80	2.13 ± 0.96	2.10 ± 0.85	−0.03 ± 0.69	0.871

Abbreviations: BCAA, branched-chain amino acids (the sum of l-valine, l-leucine, and l-isoleucine); CAA, competing amino acids (the sum of the BCAA, l-phenylalanine, and l-tyrosine); l-Trp; l-tryptophan; 24 h, 24 h; Δ cortisol; cortisol difference morning-evening; SD, standard deviation; ^1^ Intention to treat (ITT) population; *p*-Value: **, *p* < 0.001; *, *p* < 0.05 (Wilcoxon test); †, significant group difference at baseline; ^a^ Mann–Whitney U test; ^b^ data not available for one participant in the placebo group at baseline (hemolytic blood sample).

**Table 5:** Changes in anthropometric, clinical, and cardiometabolic parameters during the dietary intervention.

	Verum Group (*n* = 29) ^1^			Placebo Group (*n* = 30) ^1^			V vs. P
	Mean ± SD; *n* (%)			Mean ± SD; *n* (%)			*p*-Value ^a^
	Baseline	Week 12	Difference	Baseline	Week 12	Difference	12 Weeks
Weight (kg)	73.3 ± 13.3	73.5 ± 13.3	0.2 ± 1.2	76.2 ± 21.4	77.1 ± 22.4	0.9 ± 2.0*	0.292
Body mass index (BMI, kg/m²)	25.2 ± 4.4	25.3 ± 4.3	0.1 ± 0.4	25.9 ± 5.8	26.2 ± 6.1	0.3 ± 0.6*	0.253
Waist circumference (WC, cm)	90.2 ± 12.5	90.3 ± 12.4	0.1 ± 1.3	92.1 ± 16.5	92.8 ± 16.6	0.7 ± 1.8*	0.133
Waist-to-height ratio (WHtR)	0.530 ± 0.077	0.531 ± 0.076	0.001 ± 0.007	0.540 ± 0.085	0.543 ± 0.085	0.004 ± 0.011*	0.120
Systolic blood pressure (mmHg)	113.7 ± 12.6	111.0 ± 11.3	−2.7 ± 7.2 *	111.2 ± 15.8	108.6 ± 14.9	−2.5 ± 7.3*	0.871
Diastolic blood pressure (mmHg)	75.1 ± 8.2	73.8 ± 6.9	−1.3 ± 5.1	75.7 ± 9.0	74.5 ± 8.5	−1.2 ± 4.7	0.973
Resting heart rate (1/min)	66.7 ± 9.0	65.3 ± 7.4	−1.4 ± 6.8	66.5 ± 8.4	67.4 ± 10.0	1.0 ± 9.0	0.495
Resting heart rate (female; V: *n* = 21, P: *n* = 20)	67.8 ± 9.1	67.3 ± 6.9	−0.5 ± 7.2	66.2 ± 8.4	65.2 ± 8.6	−1.0 ± 8.4	0.412
Resting heart rate (male; V: *n* = 8, P: *n* = 10)	64.0 ± 8.8	60.2 ± 6.5	−3.8 ± 5.0	67.0 ± 8.9	71.9 ± 11.6	4.9 ± 9.3	0.011
Pulse pressure (mmHg)	38.6 ± 7.2	37.2 ± 6.1	−1.4 ± 5.1	35.5 ± 9.2	34.2 ± 8.7	−1.3 ± 5.8	0.919
Total cholesterol (TC, mg/dL)	209.9 ± 51.7	206.4 ± 47.3	−3.4 ± 20.6	213.1 ± 43.2	210.9 ± 45.7	−2.2 ± 25.7	0.931
HDL-C (mg/dL)	64.3 ± 18.6	65.8 ± 19.0	1.6 ± 7.2	64.4 ± 21.7	63.8 ± 23.4	−0.6 ± 8.0	0.203
LDL-C (mg/dL)	130.1 ± 48.0	125.9 ± 43.4	−4.2 ± 18.6	137.2 ± 35.1	132.6 ± 35.8	−4.6 ± 21.8	0.961
Triglycerides (TG, mg/dL)	109.2 ± 64.9	109.7 ± 63.7	0.5 ± 35.5	92.7 ± 41.8	110.7 ± 92.2	18.0 ± 75.6	0.606
Fasting plasma glucose (FPG, mg/dL)	88.1 ± 9.3	86.2 ± 7.2	−1.8 ± 6.2	91.4 ± 22.6	94.5 ± 35.0	3.1 ± 15.5	0.295
HbA1_c_ (glycated hemoglobin A1_c_, %)	5.41 ± 0.29	5.41 ± 0.24	0.00 ± 0.30	5.63 ± 0.75	5.62 ± 0.94	−0.01 ± 0.29	0.841
HOMA-index	1.50 ± 0.84	1.50 ± 1.05	0.01 ± 0.91	1.96 ± 2.04	2.83 ± 4.89	0.87 ± 3.47	0.859
Insulin-ECLIA (µU/mL)	6.75 ± 3.54	7.02 ± 4.71	0.27 ± 4.06	7.83 ± 4.90	10.00 ± 13.71	2.17 ± 11.49	0.839
C-reactive protein sensitive (CRP, mg/L)	2.86 ± 3.89	2.23 ± 3.23	−0.62 ± 3.46	1.65 ± 2.97	1.85 ± 2.46	0.21 ± 3.48	0.312
Ferritin (ng/mL)	99.2 ± 68.5	80.9 ± 81.2	−18.3 ± 23.4 **	129.1 ± 135.5	145.8 ± 182.0	16.7 ± 107.0	0.011
Gamma-glutamyltransferase (GGT, U/L)	21.7 ± 14.8	23.7 ± 20.2	1.9 ± 13.0	22.8 ± 12.2	24.4 ± 20.9	1.6 ± 18.1	0.088
Aspartate aminotransferase (ASAT, U/L)	27.0 ± 17.6	26.3 ± 5.9	−0.8 ± 14.7	23.5 ± 4.7	35.5 ± 66.0	12.0 ± 66.3	0.126
Alanine aminotransferase (ALAT, U/L)	23.9 ± 12.4	27.3 ± 15.4	3.4 ± 8.1 *	23.3 ± 12.1	52.7 ± 162.5	29.4 ± 163.4	0.047
GFR (CKD-EPI) (mL/min)	96.2 ± 16.3	97.3 ± 15.0	1.1 ± 5.8	95.8 ± 13.1	97.7 ± 14.2	1.8 ± 8.0	0.830
Fasting plasma glucose (FPG) ≥ 100 mg/dL	3 (10.3%)	2 (6.9%)	−1 (−33%)	4 (13.3%)	7 (23.3%)	3 (75%)	0.343
Metabolic syndrome	3 (10.3%)	2 (6.9%)	−1 (−33%)	3 (10%)	6 (20%)	3 (100%)	0.531

Abbreviations: ECLIA, electrochemiluminescence immunoassay; GFR (CKD-EPI), glomerular filtration rate (Chronic Kidney Disease Epidemiology Collaboration); HDL-C, high-density lipoprotein cholesterol; HOMA-index, homeostasis model assessment index; LDL-C, low-density lipoprotein cholesterol; SD, standard deviation; ^1^ Intention to treat (ITT) population; *p*-Value: **, *p* < 0.001; *, *p* < 0.05 (Wilcoxon test); ^a^ Mann–Whitney U test or Fisher’s exact test.

## Supplementary Materials

The following are available online at http://www.mdpi.com/2072-6643/10/5/551/s1: Table S1: Values of the PSQ_30_ before and during the dietary intervention, Table S2: Values of the PNF before and after the dietary intervention, Table S3: Values of the VAS before and during the dietary intervention, Table S4: Changes in salivary cortisol, serotonin, mineral, and vitamin concentrations during the dietary intervention, Table S5: Changes in serum amino acid concentrations during the dietary intervention, Table S6: Changes in urinary concentrations during the dietary intervention, Table S7: Changes in anthropometric and clinical parameters during the dietary intervention, Table S8: Changes in cardiometabolic parameters during the dietary intervention, Table S9: Changes in further serum parameters during the dietary intervention; Table S10: Linear regression analysis; Table S11: Dietary intake at baseline and after the dietary intervention.
