# Supplementary material for: Impact of a Specific Amino Acid Composition with Micronutrients on Well-Being in Subjects with Chronic Psychological Stress and Exhaustion Conditions: A Pilot Study
Source: Nutrients. 2018 Apr 29;10(5):551. doi: 10.3390/nu10050551 (PMC5986431; doi:10.3390/nu10050551)
Supplement: Supplementary file 1 [file nutrients-10-00551-s001.zip › Tables S1-S9. Medians and interquartile ranges of the outcome measures.pdf]

**Table S1.** Values of the $PSQ_{30}$ before and during the dietary intervention

| | Verum group (*n* = 29)[1]<br>mean ± SD<br>median<br>(Q1/Q3) | | | | | Placebo group (*n* = 30)[1]<br>mean ± SD<br>median<br>(Q1/Q3) | | | | | V vs. P<br>*p*-Value [b] | |
|---|---|---|---|---|---|---|---|---|---|---|---|---|
| | **Baseline** | **Week 8** | **Week 12** | **Difference 8 weeks** | **Difference 12 weeks** | **Baseline** | **Week 8** | **Week 12** | **Difference 8 weeks** | **Difference 12 weeks** | **8 weeks** | **12 weeks** |
| **Total $PSQ_{30}$ score** | 0.67 ± 0.12<br>0.66<br>(0.56/0.79) | 0.54 ± 0.16<br>0.51<br>(0.44/0.69) | 0.48 ± 0.16<br>0.47<br>(0.35/0.59) | -0.13 ± 0.14**<br>-0.11<br>(-0.21/-0.04) | -0.19 ± 0.16**<br>-0.18<br>(-0.32/-0.06) | 0.66 ± 0.12<br>0.68<br>(0.53/0.75) | 0.607 ± 0.127<br>0.600<br>(0.54/0.71) | 0.58 ± 0.16<br>0.57<br>(0.45/0.70) | -0.06 ± 0.12*<br>-0.07<br>(-0.14/0.03) | -0.08 ± 0.12**<br>-0.09<br>(-0.16/-0.01) | 0.092 | 0.012 |
| Harassment | 2.8 ± 0.5<br>2.8<br>(2.2/3.2) | 2.4 ± 0.5<br>2.5<br>(2.0/2.8) | 2.2 ± 0.6<br>2.0<br>(1.8/2.5) | -0.4 ± 0.6*<br>-0.5<br>(-0.8/0.0) | -0.6 ± 0.7**<br>-0.5<br>(-1.1/0.0) | 2.8 ± 0.5<br>2.8<br>(2.4/3.1) | 2.6 ± 0.6<br>2.5<br>(2.2/3.0) | 2.5 ± 0.6<br>2.5<br>(2.0/3.1) | -0.2 ± 0.6<br>-0.2<br>(-0.5/0.0) | -0.3 ± 0.6*<br>-0.4<br>(-0.8/0.1) | 0.370 | 0.067 |
| Overload | 3.1 ± 0.5<br>3.2<br>(2.8/3.5) | 2.7 ± 0.6<br>2.5<br>(2.2/3.1) | 2.6 ± 0.7<br>2.5<br>(2.1/3.0) | -0.4 ± 0.4**<br>-0.5<br>(-0.8/0.0) | -0.5 ± 0.5**<br>-0.5<br>(-1.0/0.0) | 3.1 ± 0.5<br>3.1<br>(2.8/3.5) | 3.0 ± 0.5<br>3.0<br>(2.5/3.3) | 2.9 ± 0.7<br>(2.9<br>(2.5/3.5) | -0.1 ± 0.5<br>-0.2<br>(-0.5/0.1) | -0.2 ± 0.5*<br>0.0<br>(-0.5/0.0) | 0.046 | 0.014 |
| Irritability | 3.0 ± 0.6<br>3.0<br>(2.8/3.5) | 2.7 ± 0.7<br>2.5<br>(2.0/3.2) | 2.4 ± 0.6<br>2.5<br>(2.0/3.0) | -0.3 ± 0.7*<br>-0.5<br>(-1.0/0.0) | -0.6 ± 0.7**<br>-1.0<br>(-1.0/0.0) | 2.8 ± 0.6<br>3.0<br>(2.5/3.5) | 2.8 ± 0.6<br>3.0<br>(2.5/3.0) | 2.6 ± 0.6<br>2.5<br>(2.4/3.0) | 0.0 ± 0.6<br>0.0<br>(-0.5/0.5) | -0.2 ± 0.6*<br>0.0<br>(-0.5/0.0) | 0.143 | 0.030 |
| Lack of joy | 3.0 ± 0.4<br>3.0<br>(2.8/3.4) | 2.7 ± 0.5<br>2.9<br>(2.3/3.1) | 2.6 ± 0.4<br>2.6<br>(2.2/2.9) | -0.3 ± 0.5*<br>-0.3<br>(-0.8/0.1) | -0.4 ± 0.4**<br>-0.4<br>(-0.6/-0.1) | 3.0 ± 0.5<br>3.1<br>(2.7/3.4) | 2.9 ± 0.4<br>2.9<br>(2.7/3.3) | 2.9 ± 0.6<br>2.8<br>(2.5/3.3) | -0.1 ± 0.3<br>-0.2<br>(-0.3/0.1) | -0.1 ± 0.4*<br>-0.1<br>(-0.3/0.1) | 0.199 | 0.006 |
| Fatigue | 3.3 ± 0.4<br>3.2<br>(3.0/3.8) | 2.9 ± 0.6<br>3.0<br>(2.5/3.4) | 2.7 ± 0.5<br>2.8<br>(2.2/3.0) | -0.4 ± 0.7*<br>-0.2<br>(-0.8/0.0) | -0.6 ± 0.6**<br>-0.5<br>(-0.9/-0.2) | 3.3 ± 0.4<br>3.2<br>(3.0/3.6) | 3.0 ± 0.5<br>3.2<br>(2.8/3.5) | 3.0 ± 0.5<br>3.0<br>(2.5/3.5) | -0.2 ± 0.4*<br>-0.2<br>(-0.5/0.0) | -0.3 ± 0.5*<br>-0.2<br>(-0.8/0.0) | 0.339 | 0.034 |
| Worries | 2.8 ± 0.6<br>2.8<br>(2.2/3.4) | 2.3 ± 0.7<br>2.2<br>(1.8/2.9) | 2.2 ± 0.6<br>2.0<br>(1.8/2.8) | -0.5 ± 0.5**<br>-0.6<br>(-0.8/0.0) | -0.6 ± 0.6**<br>-0.6<br>(-1.0/-0.2) | 2.8 ± 0.4<br>2.8<br>(2.6/3.2) | 2.6 ± 0.4<br>2.6<br>(2.4/3.0) | 2.5 ± 0.6<br>2.4<br>(2.0/3.0) | -0.2 ± 0.4*<br>-0.2<br>(-0.4/0.0) | -0.4 ± 0.5**<br>-0.4<br>(-0.6/-0.2) | 0.121 | 0.140 |
| Tension | 3.1 ± 0.4<br>3.0<br>(2.8/3.5) | 2.6 ± 0.6<br>2.5<br>(2.0/3.0) | 2.4 ± 0.7<br>2.2<br>(2.0/2.8) | -0.5 ± 0.5**<br>-0.5<br>(-0.8/-0.2) | -0.7 ± 0.6**<br>-0.8<br>(-1.2/-0.1) | 3.0 ± 0.5<br>3.0<br>(2.5/3.3) | 2.8 ± 0.6<br>2.8<br>(2.2/3.1) | 2.7 ± 0.6<br>2.6<br>(2.2/3.1) | -0.2 ± 0.6<br>-0.2<br>(-0.6/0.2) | -0.3 ± 0.6*<br>-0.2<br>(-0.8/0.2) | 0.077 | 0.012 |

Abbreviations: PSQ, Perceived Stress Questionnaire; SD, standard deviation; Q1/Q3, first quartile/third quartile; [1]Intention to treat (ITT) population; *p*-Value: **, $p < 0.001$; *, $p < 0.05$ (Wilcoxon-test within groups), [b] Mann-Whitney U test.

**Table S2.** Values of the PNF before and after the dietary intervention

| | **Verum group ($n$ = 29)[1] mean ± SD median (Q1/Q3)** | | | | | **Placebo group ($n$ = 30)[1] mean ± SD median (Q1/Q3)** | | | | | **V vs. P $p$-Value [b]** | |
|---|---|---|---|---|---|---|---|---|---|---|---|---|
| | **Baseline** | **Week 8** | **Week 12** | **Difference 8 weeks** | **Difference 12 weeks** | **Baseline** | **Week 8** | **Week 12** | **Difference 8 weeks** | **Difference 12 weeks** | **8 weeks** | **12 weeks** |
| **Total PNF points** | 53.7 ± 13.2<br>55.0<br>(43.5/62.0) | [a] | 39.3 ± 14.4<br>36.0<br>(29.0/47.5) | - | -14.3 ± 11.8**<br>-14.0<br>(-21.0/-6.5) | 48.3 ± 10.4<br>48.5<br>(39.8/54.2) | [a] | 41.3 ± 14.6<br>41.5<br>(29.5/52.8) | - | -7.0 ± 12.5*<br>-8.0<br>(-15.2/-0.8) | - | 0.063 |
| Psycho-neuro-vegetative stability | 19.0 ± 5.2<br>19.0<br>(15.0/23.0) | [a] | 13.3 ± 4.7<br>12.0<br>(10.0/16.5) | - | -5.7 ± 4.7**<br>-6.0<br>(-8.5/-2.0) | 17.9 ± 4.4<br>18.0<br>(14.0/21.0) | [a] | 14.6 ± 5.2<br>15.0<br>(11.0/17.2) | - | -3.3 ± 5.2*<br>-4.0<br>(-7.0/-0.8) | - | 0.136 |
| Neurological symptoms | 7.2 ± 3.7<br>7.0<br>(5.0/9.0) | [a] | 4.6 ± 2.9<br>4.0<br>(2.5/6.5) | - | -2.6 ± 2.5**<br>-2.0<br>(-4.5/-1.0) | 6.3 ± 3.5<br>6.0<br>(4.0/8.2) | [a] | 4.8 ± 3.4<br>4.0<br>(3.0/6.2) | - | -1.5 ± 3.2*<br>-1.5<br>(-4.0/0.0) | - | 0.246 |
| Impulsion | 11.8 ± 3.7<br>12.0<br>(9.0/14.5) | [a] | 8.4 ± 4.5<br>8.0<br>(5.5/12.0) | - | -3.4 ± 3.6**<br>-3.0<br>(-5.5/-1.0) | 9.8 ± 3.3<br>10.0<br>(7.8/12.0) | [a] | 9.1 ± 4.1<br>8.0<br>(6.0/13.0) | - | -0.7 ± 3.4<br>-1.5<br>(-3.2/1.2) | - | 0.010 |
| Excitability | 5.7 ± 2.9<br>5.0<br>(4.0/8.0) | [a] | 4.5 ± 2.9<br>4.0<br>(2.0/6.0) | - | -1.2 ± 2.2*<br>-1.0<br>(-3.0/0.5) | 4.9 ± 2.0<br>5.0<br>(4.0/6.2) | [a] | 4.3 ± 2.4<br>4.0<br>(2.0/6.2) | - | -0.6 ± 2.0<br>0.0<br>(-2.0/1.0) | - | 0.334 |
| Concentration and memory | 9.9 ± 3.7<br>11.0<br>(6.5/12.5) | [a] | 8.5 ± 3.9<br>8.0<br>(6.0/9.5) | - | -1.4 ± 3.0*<br>-1.0<br>(-4.0/0.5) | 9.4 ± 2.9<br>9.0<br>(7.0/12.2) | [a] | 8.5 ± 3.2<br>8.5<br>(6.0/11.2) | - | -0.9 ± 2.9<br>-0.5<br>(-3.2/1.0) | - | 0.507 |

Abbreviations: PNF, Psychological Neurological Questionnaire; SD, standard deviation; Q1/Q3, first quartile/third quartile; [1]Intention to treat (ITT) population; [a] not implemented according to the study protocol; $p$-Value: **, $p < 0.001$; *, $p < 0.05$ (Wilcoxon- test within groups), [b] Mann-Whitney U test.

**Table S3.** Values of the VAS before and during the dietary intervention

| | Verum group (*n* = 29)[1] mean ± SD median (Q1/Q3) | | | | | Placebo group (*n* = 30)[1] mean ± SD median (Q1/Q3) | | | | | V vs. P *p*-Value [b] | |
|---|---|---|---|---|---|---|---|---|---|---|---|---|
| | **Baseline** | **Week 8** | **Week 12** | **Difference 8 weeks** | **Difference 12 weeks** | **Baseline** | **Week 8** | **Week 12** | **Difference 8 weeks** | **Difference 12 weeks** | **8 weeks** | **12 weeks** |
| **Total VAS points** | 15.9 ± 3.6<br>16.0<br>(14.0/18.0) | 14.1 ± 3.7<br>14.0<br>(12.0/16.0) | 12.7 ± 4.4<br>14.0<br>(8.5/16.0) | -1.8 ± 3.9<br>-2.0<br>(-4.5/0.5) | -3.3 ± 4.2**<br>-2.0<br>(-6.0/-1.0) | 15.3 ± 3.6<br>15.5<br>(12.8/17.2) | 15.0 ± 3.8<br>15.0<br>(12.8/17.2) | 14.5 ± 4.3<br>15.0<br>(10.0/17.5) | -0.3 ± 4.6<br>-1.0<br>(-4.0/3.2) | -0.8 ± 5.5<br>-2.0<br>(-5.0/3.0) | 0.284 | 0.178 |
| Lack of motivation | 3.1 ± 1.0<br>3.0<br>(2.5/4.0) | 2.7 ± 0.8<br>3.0<br>(2.0/3.0) | 2.4 ± 1.1<br>2.0<br>(1.5/3.0) | -0.4 ± 1.1<br>-1.0<br>(-1.0/0.5) | -0.7 ± 1.1*<br>-1.0<br>(-1.0/0.0) | 3.2 ± 0.9<br>3.0<br>(3.0/4.0) | 2.9 ± 1.0<br>3.0<br>(2.0/4.0) | 2.9 ± 1.0<br>3.0<br>(2.0/4.0) | -0.3 ± 1.2<br>0.0<br>(-1.0/0.2) | -0.4 ± 1.3<br>-1.0<br>(-1.0/0.2) | 0.671 | 0.558 |
| Indifference | 3.0 ± 1.0<br>3.0<br>(2.5/4.0) | 2.4 ± 1.1<br>2.0<br>(2.0/3.5) | 2.2 ± 1.0<br>2.0<br>(1.0/3.0) | -0.5 ± 1.3*<br>0.0<br>(-1.0/0.0) | -0.8 ± 1.1*<br>0.0<br>(-2.0/0.0) | 2.8 ± 1.0<br>3.0<br>(2.0/4.0) | 2.8 ± 1.0<br>3.0<br>(2.0/3.0) | 2.5 ± 1.1<br>2.0<br>(2.0/3.0) | -0.0 ± 1.2<br>0.0<br>(-1.0/1.0) | -0.3 ± 1.5<br>-0.5<br>(-2.0/1.0) | 0.155 | 0.235 |
| Fatigue | 3.4 ± 1.1<br>3.0<br>(3.0/4.0) | 3.1 ± 1.1<br>3.0<br>(3.0/4.0) | 2.8 ± 1.2<br>3.0<br>(2.0/4.0) | -0.3 ± 1.3<br>0.0<br>(-1.0/1.0) | -0.6 ± 1.3*<br>0.0<br>(-1.5/0.0) | 3.5 ± 1.0<br>4.0<br>(3.0/4.0) | 3.4 ± 1.1<br>3.0<br>(3.0/4.0) | 3.5 ± 1.0<br>3.0<br>(3.0/4.2) | -0.1 ± 1.4<br>0.0<br>(-1.0/1.0) | 0.0 ± 1.3<br>0.0<br>(-1.0/1.0) | 0.728 | 0.096 |
| Mood changes | 3.2 ± 0.9<br>3.0<br>(3.0/4.0) | 2.9 ± 1.0<br>3.0<br>(2.0/3.5) | 2.7 ± 1.0<br>3.0<br>(2.0/3.0) | -0.3 ± 1.1<br>0.0<br>(-1.0/0.0) | -0.5 ± 1.1*<br>0.0<br>(-1.0/0.0) | 2.8 ± 1.0<br>3.0<br>(2.0/4.0) | 2.9 ± 1.1<br>3.0<br>(2.0/4.0) | 2.6 ± 1.1<br>2.0<br>(2.0/3.0) | 0.1 ± 1.2<br>0.0<br>(-1.0/1.0) | -0.2 ± 1.5<br>0.0<br>(-1.0/1.0) | 0.250 | 0.389 |
| Productivity | 3.1 ± 1.1<br>3.0<br>(2.0/4.0) | 2.9 ± 0.8<br>3.0<br>(2.0/3.0) | 2.5 ± 1.0<br>2.0<br>(2.0/3.0) | -0.2 ± 0.9<br>0.0<br>(-1.0/0.0) | -0.6 ± 1.1*<br>-1.0<br>(-1.0/0.0) | 2.9 ± 0.9<br>3.0<br>(2.8/3.2) | 3.0 ± 1.0<br>3.0<br>(2.0/4.0) | 3.0 ± 0.9<br>3.0<br>(2.0/4.0) | 0.1 ± 1.2<br>0.0<br>(-1.0/1.0) | 0.0 ± 1.2<br>0.0<br>(-1.0/1.0) | 0.364 | 0.040 |

Abbreviations: VAS, visual analogue scales; SD, standard deviation; Q1/Q3, first quartile/third quartile; [1]Intention to treat (ITT) population; *p*-Value: **, $p < 0.001$; *, $p < 0.05$ (Wilcoxon- test within groups), [b] Mann-Whitney U test.

**Table S4.** Changes in salivary cortisol, serotonin, mineral, and vitamin concentrations during the dietary intervention

| | **Verum group (*n* = 29)[1] Mean ± SD Median (Q1/Q3)** | | | **Placebo group (*n* = 30)[1] Mean ± SD Median (Q1/Q3)** | | | **V vs. P *p*-Value [a]** |
|---|---|---|---|---|---|---|---|
| | **Baseline** | **Week 12** | **Difference** | **Baseline** | **Week 12** | **Difference** | **12 weeks** |
| **Salivary** | | | | | | | |
| Cortisol_morning (ng/ml) | 12.54 ± 6.61 12.2 (3.2/25.4) | 13.96 ± 5.76 13.3 (5.3/26.7) | 1.42 ± 8.17 1.4 (-14.9/15.4) | 14.62 ± 4.55 14.6 (5.0/21.5) | 15.08 ± 6.64 14.35 (4.2/27.2) | 0.47 ± 5.78 0.95 (-9.1/11.8) | 0.596 |
| Cortisol_evening (ng/ml) | 3.00 ± 4.90 1.50 (1.20/2.50) | 2.07 ± 1.80 1.70 (1.20/2.45) | -0.93 ± 5.03 -0.10 (-0.70/0.40) | 2.04 ± 2.49 1.25 (0.90/2.05) | 2.59 ± 3.60 1.50 (0.88/2.52) | 0.55 ± 4.10 -0.05 (-0.52/0.72) | 0.495 |
| Δ cortisol (m – e) (ng/ml) | 9.54 ± 6.37 9.70 (2.95/12.85) | 11.89 ± 6.05 11.20 (6.50/17.30) | 2.34 ± 7.32 1.10 (-2.25/8.80) | 12.57 ± 5.35 13.00 (9.00/16.78) | 12.49 ± 7.98 12.25 (8.02/18.40) | -0.08 ± 7.60 0.50 (-5.78/5.58) | 0.300 |
| **Serum** | | | | | | | |
| Serotonin (µg/L) | 137.7 ± 58.0 126.0 (92.0/182.5) | 138.7 ± 57.3 129.0 (87.0/186.5) | 1.0 ± 20.9 2.0 (-6.5/12.0) | 150.1 ± 80.9 136.5 (107.0/165.5) | 140.5 ± 99.8 121.0 (84.8/143.5) | -9.6 ± 32.4* -8.5 (-25.5/3.8) | 0.047 |
| Folic acid (ng/mL) [b] | 9.67 ± 4.13 8.60 (6.55/12.40) | 18.18 ± 2.84 20.00 (16.45/20.00) | 8.51 ± 4.38** 10.00 (5.75/11.50) | 9.48 ± 3.08 8.20 (7.65/10.85) | 8.93 ± 3.71 7.80 (6.28/11.05) | -0.55 ± 2.48 -0.70 (-1.75/0.75) | < 0.001 |
| Magnesium (mmol/L) | 0.87 ± 0.06 0.87 (0.84/0.90) | 0.87 ± 0.05 0.85 (0.82/0.90) | -0.00 ± 0.04 -0.01 (-0.03/0.03) | 0.89 ± 0.06 0.90 (0.85/0.94) | 0.86 ± 0.06 0.86 (0.83/0.90) | -0.03 ± 0.05* -0.04 (-0.06/0.01) | 0.021 |
| Calcium (mmol/L) | 2.38 ± 0.08 2.38 (2.33/2.43) | 2.37 ± 0.09 2.37 (2.31/2.42) | -0.01 ± 0.10 -0.02 (-0.09/0.07) | 2.40 ± 0.12 2.39 (2.30/2.51) | 2.37 ± 0.10 2.36 (2.29/2.44) | -0.03 ± 0.10* (-0.04 (-0.10/0.02) | 0.413 |

Abbreviations: Δ cortisol; cortisol difference morning-evening; SD, standard deviation; Q1/Q3, first quartile/third quartile; [1]Intention to treat (ITT) population; *p*-Value: **, $p < 0.001$; *, $p < 0.05$ (Wilcoxon test); [a] Mann-Whitney U test; [b] data not available for one participant in the placebo group at baseline (hemolytic blood sample).

**Table S5.** Changes in serum amino acid concentrations during the dietary intervention

| | **Verum group (*n* = 29)[1] Mean ± SD Median (Q1/Q3)** | | | **Placebo group (*n* = 30)[1] Mean ± SD Median (Q1/Q3)** | | | **V vs. P *p*-Value [a]** |
|---|---|---|---|---|---|---|---|
| | **Baseline** | **Week 12** | **Difference** | **Baseline** | **Week 12** | **Difference** | **12 weeks** |
| Protein (g/dL) † | 7.15 ± 0.34<br>7.07<br>(6.6/8.0) | 7.16 ± 0.40<br>7.08<br>(6.3/8.2) | 0.01 ± 0.36<br>0.0<br>(-0.8/0.8) | 7.41 ± 0.43<br>7.44<br>(6.6/8.6) | 7.24 ± 0.44<br>7.14<br>(6.4/8.3) | -0.17 ± 0.38*<br>-0.13<br>(-1.0/0.8) | 0.075 |
| L-arginine (μmol/L) † | 115.9 ± 27.9<br>113.0<br>(97.5/120.5) | 122.7 ± 25.4<br>119.0<br>(109.0/133.0) | 6.8 ± 19.4<br>8.0<br>(-6.0/18.5) | 132.7 ± 22.8<br>133.5<br>(119.2/145.0) | 126.3 ± 19.5<br>123.0<br>(113.5/142.2) | -6.4 ± 23.1<br>-8.5<br>(-19.5/11.5) | 0.014 |
| L-ornithine (μmol/L) | 106.7 ± 33.0<br>99 .0<br>(85.5/118.0) | 108.4 ± 67.4<br>103.0<br>(76.0/117.0) | 1.8 ± 52.0<br>-10.0<br>(-18.0/11.0) | 100.3 ± 21.3<br>98.5<br>(84.0/116.0) | 100.4 ± 32.5<br>97.5<br>(79.8/117.8) | 0.1 ± 22.5<br>-4.0<br>(-18.0/20.2) | 0.590 |
| L-phenylalanine (μmol/L) | 95.6 ± 19.8<br>96.0<br>(83.5/112.0) | 97.5 ± 14.5<br>96.0<br>(88.0/109.0) | 1.9 ± 17.9<br>0.0<br>(-4.5/6.0) | 99.6 ± 13.2<br>97.0<br>(89.5/109.0) | 94.9 ± 15.9<br>95.0<br>(83.8/110.0) | -4.6 ± 15.7<br>-4.5<br>(-16.2/4.2) | 0.090 |
| Taurine (μmol/L) | 157.0 ± 35.5<br>158.0<br>(126.0/187.5) | 175.3 ± 49.5<br>173.0<br>(144.0/198.5) | 18.3 ± 38.2*<br>20.0<br>(-9.0/44.0) | 154.6 ± 27.4<br>155.5<br>(133.0/168.8) | 151.4 ± 43.6<br>146.0<br>(123.8/169.5) | -3.2 ± 40.1<br>1.0<br>(-31.0/23.5) | 0.027 |
| L-Trp (μmol/L) | 53.0 ± 14.8<br>50.0<br>(43.5/58.5) | 53.1 ± 9.3<br>50.0<br>(46.0/60.2) | 0.1 ± 14.9<br>3.0<br>(-3.0/5.0) | 54.9 ± 9.4<br>54.5<br>(48.0/60.2) | 54.6 ± 9.8<br>54.0<br>(46.8/58.5) | -0.3 ± 7.8<br>-1.5<br>(-5.2/4.2) | 0.197 |
| L-tyrosine (μmol/L) | 67.5 ± 17.4<br>67.0<br>(57.0/78.0) | 71.4 ± 21.9<br>67.0<br>(62.0/76.0) | 3.9 ± 18.6<br>3.0<br>(-9.0/10.0) | 70.4 ± 13.1<br>72.0<br>(65.8/76.8) | 69.2 ± 14.7<br>66.5<br>(61.0/76.8) | -1.2 ± 19.3<br>-4.5<br>(-9.5/2.5) | 0.102 |
| L-Trp/CAA | 0.087 ± 0.033<br>0.082<br>(0.074/0.090) | 0.088 ± 0.015<br>0.086<br>(0.078/0.096) | 0.001 ± 0.033*<br>0.006<br>(0.000/0.014) | 0.086 ± 0.013<br>0.082<br>(0.075/0.096) | 0.087 ± 0.015<br>0.086<br>(0.076/0.093) | 0.001 ± 0.014<br>0.002<br>(-0.008/0.011) | 0.181 |
| L-Trp/BCAA | 0.117 ± 0.040<br>0.115<br>(0.104/0.119) | 0.122 ± 0.021<br>0.117<br>(0.110/0.132) | 0.005 ± 0.039*<br>0.011<br>(-0.003/0.025) | 0.118 ± 0.021<br>0.112<br>(0.100/0.134) | 0.118 ± 0.022<br>0.114<br>(0.099/0.130) | 0.000 ± 0.021<br>0.001<br>(-0.014/0.014) | 0.088 |

Abbreviations: BCAA, branched-chain amino acids (the sum of L-valine, L-leucine, and L-isoleucine); CAA, competing amino acids (the sum of the BCAA, L-phenylalanine, and L-tyrosine); L-Trp; L-tryptophan; SD, standard deviation; Q1/Q3, first quartile/third quartile; [1]Intention to treat (ITT) population; *p*-Value: **, $p < 0.001$; *, $p < 0.05$ (Wilcoxon test); †, significant group difference at baseline; [a] Mann-Whitney U test.

**Table S6.** Changes in urinary concentrations during the dietary intervention

| | Verum group ($n$ = 29)[1] Mean ± SD Median (Q1/Q3) | | | Placebo group ($n$ = 30)[1] Mean ± SD Median (Q1/Q3) | | | V vs. P $p$-Value [a] |
|---|---|---|---|---|---|---|---|
| | Baseline | Week 12 | Difference | Baseline | Week 12 | Difference | 12 weeks |
| Magnesium (mmol/24 h) | 3.38 ± 1.41<br>3.22<br>(2.95/4.88) | 5.67 ± 1.49<br>5.49<br>(4.64/7.10) | 1.84 ± 1.56**<br>1.59<br>(-1.08/2.97) | 3.63 ± 1.27<br>3.52<br>(2.56/4.46) | 3.84 ± 1.61<br>3.48<br>(2.80/4.77) | 0.21 ± 1.22<br>0.44<br>(-0.30/0.78) | < 0.001 |
| Calcium (mmol/24 h) | 4.88 ± 2.36<br>4.18<br>(3.33/6.08) | 5.60 ± 3.19<br>5.10<br>(3.20/6.88) | 0.71 ± 2.58*<br>0.70<br>(-0.30/1.80) | 4.58 ± 3.26<br>3.39<br>(2.62/5.97) | 4.32 ± 2.93<br>3.40<br>(2.47/5.30) | -0.26 ± 1.56<br>-0.32<br>(-1.81/0.89) | 0.023 |
| Sodium (mmol/24 h) | 166.9 ± 66.3<br>141 .0<br>(105.5/218.0) | 142.0 ± 58.1<br>137.0<br>(102.9/172.5) | -24.9 ± 65.9*<br>-20.0<br>(-80.0/29.5) | 157.4 ± 75.7<br>142.0<br>(109.4/185.0) | 172.0 ± 84.5<br>147.0<br>(122.8/217.0) | 14.7 ± 82.9<br>9.5<br>(-42.6/74.7) | 0.025 |
| Chloride (mmol/24 h) | 151.9 ± 60.8<br>135.0<br>(108.0/202.0) | 140.6 ± 56.1<br>138.0<br>(109.5/176.0) | -11.3 ± 59.1<br>-1.0<br>(-60.0/24.5) | 134.3 ± 66.4<br>126.5<br>(92.8/162.2) | 148.5 ± 76.6<br>124.5<br>(107.8/163.8) | 14.2 ± 70.3<br>19.5<br>(-31.0/67.0) | 0.145 |
| pH value | 6.22 ± 0.42<br>6.19<br>(5.90/6.59) | 6.06 ± 0.43<br>6.03<br>(5.76/6.39) | -0.16 ± 0.48<br>-0.11<br>(-0.51/0.15) | 6.16 ± 0.35<br>6.16<br>(5.92/6.38) | 6.26 ± 0.42<br>6.24<br>(5.90/6.60) | 0.10 ± 0.44<br>0.20<br>(-0.23/0.44) | 0.028 |
| Creatinine (g/24h) | 1.46 ± 0.53<br>1.34<br>(1.11/1.68) | 1.37 ± 0.44<br>1.22<br>(1.11/1.62) | -0.09 ± 0.34<br>-0.04<br>(-0.20/0.08) | 1.37 ± 0.52<br>1.21<br>(1.00/1.61) | 1.37 ± 0.53<br>1.18<br>(0.96/1.54) | -0.04 ± 0.30<br>-0.06<br>(-0.23/0.09) | 0.949 |
| Volume (L/24h) | 2.14 ± 0.69<br>2.00<br>(1.67/2.59) | 2.12 ± 0.73<br>2.00<br>(1.64/2.68) | -0.02 ± 0.80<br>-0.05<br>(-0.60/0.40) | 2.13 ± 0.96<br>2.15<br>(1.30/2.92) | 2.10 ± 0.85<br>2.20<br>(1.49/2.52) | -0.03 ± 0.69<br>-0.10<br>(-0.48/0.46) | 0.871 |

Abbreviations: SD, standard deviation; Q1/Q3, first quartile/third quartile; [1]Intention to treat (ITT) population; $p$-Value: **, $p < 0.001$; *, $p < 0.05$ (Wilcoxon test); [a] Mann-Whitney U test.

**Table S7.** Changes in anthropometric and clinical parameters during the dietary intervention

| | Verum group ($n$ = 29)[1] Mean ± SD Median (Q1/Q3) | | | Placebo group ($n$ = 30)[1] Mean ± SD Median (Q1/Q3) | | | V vs. P $p$-Value [a] |
|---|---|---|---|---|---|---|---|
| | Baseline | Week 12 | Difference | Baseline | Week 12 | Difference | 12 weeks |
| Weight (kg) | 73.3 ± 13.3<br>74.6<br>(60.8/83.9) | 73.5 ± 13.3<br>74.5<br>(61.6/83.4) | 0.2 ± 1.2<br>0.2<br>(-0.4/1.0) | 76.2 ± 21.4<br>69.6<br>(60.6/93.2) | 77.1 ± 22.4<br>69.0<br>(60.8/93.5) | 0.9 ± 2.0*<br>0.8<br>(-0.2/1.8) | 0.292 |
| Body mass index (BMI, kg/m²) | 25.2 ± 4.4<br>24.9<br>(22.1/27.0) | 25.3 ± 4.3<br>25.0<br>(22.2/27.4) | 0.1 ± 0.4<br>0.1<br>(-0.2/0.3) | 25.9 ± 5.8<br>24.3<br>(21.7/29.2) | 26.2 ± 6.1<br>24.1<br>(21.8/30.4) | 0.3 ± 0.6*<br>0.3<br>(-0.1/0.7) | 0.253 |
| Waist circumference (cm) | 90.2 ± 12.5<br>90.0<br>(81.5/99.0) | 90.3 ± 12.4<br>91.0<br>(80.5/99.0) | 0.1 ± 1.3<br>0.0<br>(-1.0/1.0) | 92.1 ± 16.5<br>88.5<br>(79.8/104.8) | 92.8 ± 16.6<br>89.0<br>(80.0/106.0) | 0.7 ± 1.8*<br>0.5<br>(0.0/1.0) | 0.133 |
| Waist-to-height ratio (WHtR) | 0.530 ± 0.077<br>0.543<br>(0.469/0.560) | 0.531 ± 0.076<br>0.543<br>(0.469/0.563) | 0.001 ± 0.007<br>0.000<br>(-0.006/0.006) | 0.540 ± 0.085<br>0.536<br>(0.481/0.580) | 0.543 ± 0.085<br>0.536<br>(0.486/0.583) | 0.004 ± 0.011*<br>0.003<br>(0.000/0.006) | 0.120 |
| Systolic blood pressure (mmHg) | 113.7 ± 12.6<br>111.0<br>(103.7/123.2) | 111.0 ± 11.3<br>109.3<br>(104.2/119.8) | -2.7 ± 7.2*<br>-2.3<br>(-6.5/0.0) | 111.2 ± 15.8<br>107.5<br>(100.8/120.5) | 108.6 ± 14.9<br>106.5<br>(98.1/120.9) | -2.5 ± 7.3*<br>-3.2<br>(-7.8/2.4) | 0.871 |
| Diastolic blood pressure (mmHg) | 75.1 ± 8.2<br>74.7<br>(69.2/81.8) | 73.8 ± 6.9<br>74.0<br>(70.3/77.8) | -1.3 ± 5.1<br>-1.3<br>(-4.5/2.3) | 75.7 ± 9.0<br>77.7<br>(69.5/80.7) | 74.5 ± 8.5<br>73.5<br>(67.1/82.3) | -1.2 ± 4.7<br>-0.8<br>(-5.1/1.8) | 0.973 |
| Resting heart rate (1/min) | 66.7 ± 9.0<br>65.7<br>(60.5/72.5) | 65.3 ± 7.4<br>65.3<br>(60.2/70.7) | -1.4 ± 6.8<br>-1.3<br>(-6.0/1.8) | 66.5 ± 8.4<br>67.0<br>(61.2/72.0) | 67.4 ± 10.0<br>67.3<br>(58.9/72.2) | 1.0 ± 9.0<br>-1.2<br>(-4.4/7.4) | 0.495 |
| Resting heart rate (female; V: n=21, P: n=20) | 67.8 ± 9.1<br>66.0<br>(61.3/73.3) | 67.3 ± 6.9<br>66.0<br>(63.0/72.7) | -0.5 ± 7.2<br>0.3<br>(-3.7/2.0) | 66.2 ± 8.4<br>66.8<br>(61.2/71.8) | 65.2 ± 8.6<br>67.0<br>(57.9/71.7) | -1.0 ± 8.4<br>-2.3<br>(-6.2/8.2) | 0.412 |
| Resting heart rate (male; V: n=8, P: n=10) | 64.0 ± 8.8<br>62.2<br>(57.7/66.8) | 60.2 ± 6.5<br>60.0<br>(56.0/64.5) | -3.8 ± 5.0<br>-4.7<br>(-6.6/1.3) | 67.0 ± 8.9<br>67.0<br>(60.1/73.2) | 71.9 ± 11.6<br>69.2<br>(64.6/77.2) | 4.9 ± 9.3<br>2.8<br>(-1.0/7.4) | 0.011 |
| Pulse pressure (mmHg) | 38.6 ± 7.2<br>39.0<br>(35.3/42.2) | 37.2 ± 6.1<br>36.3<br>(33.2/40.2) | -1.4 ± 5.1<br>-1.0<br>(-4.3/2.8) | 35.5 ± 9.2<br>33.8<br>(28.6/40.3) | 34.2 ± 8.7<br>(34.8<br>(26.5/41.1) | -1.3 ± 5.8<br>-1.0<br>(-5.3/2.0) | 0.919 |

Abbreviations: SD, standard deviation; Q1/Q3, first quartile/third quartile; [1]Intention to treat (ITT) population; $p$-Value: **, $p < 0.001$; *, $p < 0.05$ (Wilcoxon test); [a] Mann-Whitney U test.

**Table S8.** Changes in cardiometabolic parameters during the dietary intervention

| | Verum group (*n* = 29)[1] Mean ± SD Median (Q1/Q3) | | | Placebo group (*n* = 30)[1] Mean ± SD Median (Q1/Q3) | | | V vs. P *p*-Value [a] |
|---|---|---|---|---|---|---|---|
| | **Baseline** | **Week 12** | **Difference** | **Baseline** | **Week 12** | **Difference** | **12 weeks** |
| Total cholesterol (TC, mg/dl) | 209.9 ± 51.7 204.0 (180.0/228.5) | 206.4 ± 47.3 197.0 (174.5/228.5) | -3.4 ± 20.6 -5.0 (-15.5/13.0) | 213.1 ± 43.2 211.5 (178.8/247.2) | 210.9 ± 45.7 203.5 (189.2/238.5) | -2.2 ± 25.7 -2.0 (-21.2/19.0) | 0.931 |
| HDL-C (mg/dl) | 64.3 ± 18.6 64.5 (48.5/81.0) | 65.8 ± 19.0 64.0 (52.0/80.5) | 1.6 ± 7.2 1.3 (-5.0/5.6) | 64.4 ± 21.7 61.4 (46.6/75.0) | 63.8 ± 23.4 60.4 (48.8/67.9) | -0.6 ± 8.0 0.0 (-8.6/3.8) | 0.203 |
| LDL-C (mg/dl) | 130.1 ± 48.0 127.0 (94.0/156.0) | 125.9 ± 43.4 114.0 (97.5/156.5) | -4.2 ± 18.6 -8.0 (-18.0/8.0) | 137.2 ± 35.1 137.0 (108.8/164.8) | 132.6 ± 35.8 126.5 (110.5/159.0) | -4.6 ± 21.8 -8.5 (-17.5/9.0) | 0.961 |
| Triglycerides (TG, mg/dl) | 109.2 ± 64.9 87.0 (71.0/127.5) | 109.7 ± 63.7 97.0 (71.0/117.0) | 0.5 ± 35.5 5.0 (-11.5/23.0) | 92.7 ± 41.8 84.0 (71.2/105.8) | 110.7 ± 92.2 81.0 (67.8/114.5) | 18.0 ± 75.6 9.0 (-11.8/18.8) | 0.606 |
| Fasting plasma glucose (FPG, mg/dl) | 88.1 ± 9.3 85.0 (79.0/96.0) | 86.2 ± 7.2 88.0 (79.0/90.5) | -1.8 ± 6.2 0.0 (-6.5/2.0) | 91.4 ± 22.6 86.5 (79.5/95.0) | 94.5 ± 35.0 86.5 (78.8/95.5) | 3.1 ± 15.5 0.0 (-4.0/5.8) | 0.295 |
| $HbA1_c$ (glycated hemoglobin $A1_c$, %) | 5.41 ± 0.29 5.40 (5.25/5.55) | 5.41 ± 0.24 5.40 (5.20/5.60) | 0.00 ± 0.30 0.00 (-0.20/0.20) | 5.63 ± 0.75 5.45 (5.30/5.70) | 5.62 ± 0.94 5.35 (5.18/5.72) | -0.01 ± 0.29 0.00 (-0.20/0.20) | 0.841 |
| HOMA-Index | 1.50 ± 0.84 1.30 (0.85/2.05) | 1.50 ± 1.05 1.20 (0.90/1.60) | 0.01 ± 0.91 -0.10 (-0.45/0.40) | 1.96 ± 2.04 1.35 (1.05/2.10) | 2.83 ± 4.89 1.20 (0.95/1.90) | 0.87 ± 3.47 -0.10 (-0.52/0.55) | 0.859 |
| Insulin-ECLIA(μU/mL) | 6.75 ± 3.54 6.03 (3.97/8.42) | 7.02 ± 4.71 5.36 (4.19/7.21) | 0.27 ± 4.06 -0.47 (-1.92/1.96) | 7.83 ± 4.90 6.43 (4.86/8.70) | 10.00 ± 13.71 5.51 (4.72/9.42) | 2.17 ± 11.49 -0.36 (-2.26/2.56) | 0.839 |

Abbreviations: ECLIA, electrochemiluminescence immunoassay; HDL-C, high-density lipoprotein cholesterol; HOMA-index, homeostasis model assessment index; LDL-C, low-density lipoprotein cholesterol; SD, standard deviation; Q1/Q3, first quartile/third quartile; [1]Intention to treat (ITT) population; *p*-Value: **, $p < 0.001$; *, $p < 0.05$ (Wilcoxon test); [a] Mann-Whitney U test.

**Table S9.** Changes in further serum parameters during the dietary intervention

| | Verum group (*n* = 29)[1] Mean ± SD Median (Q1/Q3) | | | Placebo group (*n* = 30)[1] Mean ± SD Median (Q1/Q3) | | | V vs. P *p*-Value [a] |
|---|---|---|---|---|---|---|---|
| | **Baseline** | **Week 12** | **Difference** | **Baseline** | **Week 12** | **Difference** | **12 weeks** |
| C-reactive protein sensitive (CRP, mg/L) | 2.86 ± 3.89<br>1.40<br>(0.60/2.45) | 2.23 ± 3.23<br>1.10<br>(0.50/2.35) | -0.62 ± 3.46<br>0.00<br>(-0.75/0.35) | 1.65 ± 2.97<br>0.60<br>(0.50/1.60) | 1.85 ± 2.46<br>0.90<br>(0.40/2.55) | 0.21 ± 3.48<br>0.00<br>(-0.20/0.40) | 0.312 |
| Ferritin (ng/mL) | 99.2 ± 68.5<br>78.0<br>(44.0/152.0) | 80.9 ± 81.2<br>61.0<br>(33.0/126.0) | -18.3 ± 23.4**<br>-12.0<br>(-33.0/-2.0) | 129.1 ± 135.5<br>96.0<br>(40.2/181.8) | 145.8 ± 182.0<br>71.0<br>(35.8/180.2) | 16.7 ± 107.0<br>-2.5<br>(-13.5/15.0) | 0.011 |
| Gamma-glutamyltransferase (GGT, U/L) | 21.7 ± 14.8<br>15.0<br>(11.0/27.5) | 23.7 ± 20.2<br>16.0<br>(12.0/27.0) | 1.9 ± 13.0<br>1.0<br>(-1.5/3.0) | 22.8 ± 12.2<br>20.0<br>(12.0/32.0) | 24.4 ± 20.9<br>16.0<br>(11.8/31.2) | 1.6 ± 18.1<br>-1.0<br>(-4.0/2.0) | 0.088 |
| Aspartate aminotransferase (ASAT, U/L) | 27.0 ± 17.6<br>24.0<br>(20.0/28.0) | 26.3 ± 5.9<br>27.0<br>(22.5/29.5) | -0.8 ± 14.7<br>1.0<br>(-1.0/4.0) | 23.5 ± 4.7<br>23.5<br>(21.0/27.0) | 35.5 ± 66.0<br>22.0<br>(19.0/28.0) | 12.0 ± 66.3<br>0.0<br>(-3.0/2.0) | 0.126 |
| Alanine aminotransferase (ALAT, U/L) | 23.9 ± 12.4<br>22.0<br>(16.5/27.0) | 27.3 ± 15.4<br>22.0<br>(19.0/31.5) | 3.4 ± 8.1*<br>3.0<br>(-1.0/8.0) | 23.3 ± 12.1<br>20.5<br>(13.8/29.2) | 52.7 ± 162.5<br>18.5<br>(14.8/29.8) | 29.4 ± 163.4<br>-0.5<br>(-4.0/3.2) | 0.047 |
| GFR (CKD-EPI) (mL/min) | 96.2 ± 16.3<br>94.0<br>(83.5/106.5) | 97.3 ± 15.0<br>99.0<br>(85.5/107.5) | 1.1 ± 5.8<br>1.0<br>(-3.0/5.5) | 95.8 ± 13.1<br>95.5<br>(85.5/104.5) | 97.7 ± 14.2<br>98.5<br>(85.8/107.5) | 1.8 ± 8.0<br>1.0<br>(-4.0/7.2) | 0.830 |

Abbreviations: GFR (CKD-EPI), glomerular filtration rate (Chronic Kidney Disease Epidemiology Collaboration); SD, standard deviation; Q1/Q3, first quartile/third quartile; [1]Intention to treat (ITT) population; *p*-Value: **, $p < 0.001$; *, $p < 0.05$ (Wilcoxon test); [a] Mann-Whitney U test.
