# Supplementary material for: Impact of a Specific Amino Acid Composition with Micronutrients on Well-Being in Subjects with Chronic Psychological Stress and Exhaustion Conditions: A Pilot Study
Source: Nutrients. 2018 Apr 29;10(5):551. doi: 10.3390/nu10050551 (PMC5986431; doi:10.3390/nu10050551)
Supplement: Supplementary file 1 [file nutrients-10-00551-s001.zip › Table S10. Linear regression analysis.pdf]

Table S10. The total PSQ_{30} score, HOMA index, salivary cortisol, and CRP concentrations depending on different outcome measurements

	Group	Difference	RCB	*p*-Value
**Total PSQ_{30} score depending on (age-adjusted):**				
Total PNF points	Verum (*n* = 29)	-14.3 ± 11.8	0.010	< 0.001
	Placebo (*n* = 30)	-7.0 ± 12.5	0.006	0.001
Total VAS points	Verum (*n* = 29)	-3.3 ± 4.2	0.018	0.010
	Placebo (*n* = 30)	-0.8 ± 5.5	0.012	0.002
Δ cortisol (m – e) (ng/mL)	Verum (*n* = 29)	2.34 ± 7.32	0.006	0.143
	Placebo (*n* = 30)	-0.08 ± 7.60	0.002	0.609
Energy intake (kcal)	Verum (*n* = 29)	-17.6 ± 306.3	-0.0001	0.191
	Placebo (*n* = 30)	24.7 ± 442.5	0.0001	0.018
Systolic blood pressure (mmHg)	Verum (*n* = 29)	-2.7 ± 7.2	0.002	0.703
	Placebo (*n* = 30)	-2.5 ± 7.3	0.005	0.136
**HOMA-index depending on (age-adjusted):**				
Body weight (kg)	Verum (*n* = 29)	0.2 ± 1.2	0.177	0.208
	Placebo (*n* = 30)	0.9 ± 2.0	0.939	0.002
Resting heart rate (1/min)	Verum (*n* = 29)	-1.4 ± 6.8	0.024	0.358
	Placebo (*n* = 30)	1.0 ± 9.0	0.227	0.001
Total PSQ_{30} score	Verum (*n* = 29)	-0.192 ± 0.161	0.805	0.462
	Placebo (*n* = 30)	-0.083 ± 0.121	1.146	0.835
Folic acid (ng/mL)	Verum (*n* = 29)	8.51 ± 4.38	-0.006	0.882
	Placebo (*n* = 29)	-0.55 ± 2.48	-0.531	0.046
**Salivary cortisol in the morning (ng/mL) depending on (age-adjusted):**				
Serotonin (µg/L)	Verum (*n* = 29)	1.0 ± 20.9	0.024	0.758
	Placebo (*n* = 30)	-9.6 ± 32.4	0.070	0.037
Resting heart rate (1/min)	Verum (*n* = 29)	-1.4 ± 6.8	-0.157	0.507
	Placebo (*n* = 30)	1.0 ± 9.0	-0.010	0.934
**CRP (mg/L) depending on (age-adjusted):**				
Magnesium (mmol/L)	Verum (*n* = 29)	-0.00 ± 0.04	-33.3	0.040
	Placebo (*n* = 30)	-0.03 ± 0.05	-22.7	0.087
L-phenylalanine (µmol/L)	Verum (*n* = 29)	1.9 ± 17.9	-0.077	0.031
	Placebo (*n* = 30)	-4.6 ± 15.7	0.089	0.031

Abbreviations: CRP, C-reactive protein sensitive; HOMA-index, homeostasis model assessment index; PNF, Psychological Neurological Questionnaire; PSQ, Perceived Stress Questionnaire; RCB, regression coefficient B; SD, standard deviation; VAS, Visual Analogue Scales
