# Supplementary material for: Impact of a Specific Amino Acid Composition with Micronutrients on Well-Being in Subjects with Chronic Psychological Stress and Exhaustion Conditions: A Pilot Study
Source: Nutrients. 2018 Apr 29;10(5):551. doi: 10.3390/nu10050551 (PMC5986431; doi:10.3390/nu10050551)
Supplement: Supplementary file 1 [file nutrients-10-00551-s001.zip › Table S11. Dietary intake at baseline and after the dietary intervention.pdf]

Table S11. Dietary intake at baseline and after the dietary intervention

	**Verum group (n = 29)[1]** mean ± SD			**Placebo group (n = 30)[1]** mean ± SD			**V vs. P *p*-Value**
	**Baseline**	**Week 12**	**Difference**	**Baseline**	**Week 12**	**Difference**	**12 weeks [a]**
Energy (kcal)	2346 ± 620	2328 ± 603	-18 ± 306	2307 ± 697	2332 ± 698	25 ± 442	0.958
Protein (g)	93.7 ± 34.9	93.0 ± 28.7	-0.7 ± 23.3	87.7 ± 31.7	91.7 ± 34.2	4.0 ± 30.3	0.726
Carbohydrates (g)	244.7 ± 72.3	252.5 ± 82.5	7.9 ± 52.4	231.6 ± 65.5	243.5 ± 72.5	11.9 ± 55.5	0.934
Fat (g)	98.8 ± 32.9	93.6 ± 35.2	-5.1 ± 28.3	103.0 ± 41.8	97.6 ± 37.9	-5.4 ± 24.8	0.946
MFA (g)	33.9 ± 12.1	32.1 ± 12.4	-1.8 ± 10.4	36.3 ± 14.6	32.8 ± 14.7	-3.5 ± 10.4	0.378
SFA (g)	41.8 ± 14.9	38.0 ± 13.8	-3.8 ± 12.4	44.1 ± 21.0	43.7 ± 17.4	-0.3 ± 14.1	0.384
PFA (g)	15.7 ± 6.7	16.8 ± 10.7	1.07 ± 9.9	15.4 ± 8.0	14.2 ± 7.5	-1.2 ± 5.6	0.346
Dietary fibers (g)	21.7 ± 10.6	24.7 ± 11.0	3.0 ± 5.3*	21.8 ± 8.5	19.5 ± 6.1	-2.3 ± 6.4	0.001
Cholesterol (mg)	450.2 ± 250.6	356.5 ± 192.5	-93.7 ± 237.4*	383.8 ± 208.6	415.6 ± 223.3	31.8 ± 147.6	0.003
ß-carotene (µg)	5160 ± 4867	8840 ± 14222	3680 ± 11931	4261 ± 3782	2818 ± 2544	-1443 ± 4745	0.061
α-tocopherol (mg)	14.7 ± 7.7	15.8 ± 11.2	1.3 ± 11.4	12.8 ± 6.2	11.0 ± 5.4	-1.8 ± 5.0*	0.388
Vitamin B_1 (mg)	1.39 ± 0.88	1.37 ± 0.54	-0.02 ± 0.57	1.33 ± 0.60	1.25 ± 0.51	-0.08 ± 0.55	0.125
Vitamin B_2 (mg)	1.80 ± 0.85	1.76 ± 0.53	-0.04 ± 0.54	1.65 ± 0.60	1.70 ± 0.56	0.05 ± 0.53	0.510
Vitamin B_{12} (µg)	5.93 ± 3.38	6.09 ± 3.61	0.16 ± 2.35	6.19 ± 3.8	6.40 ± 4.21	0.21 ± 3.54	0.590
Vitamin C (mg)	129.9 ± 103.7	172.8 ± 140.3	42.9 ± 86.9*	107.4 ± 53.1	94.6 ± 63.4	-12.8 ± 65.5	0.006
Folic acid (µg)	290.0 ± 139.7	294.6 ± 104.0	4.6 ± 96.1	250.8 ± 114.1	226.2 ± 85.3	-24.6 ± 108.3	0.316
Magnesium (mg)	372.0 ± 109.6	399.5 ± 177.9	27.5 ± 90.3	374.8 ± 154.1	356.4 ± 120.3	-18.4 ± 88.7	0.053
Calcium (mg)	1029 ± 444	1071 ± 284	42 ± 349	921 ± 347	958 ± 331	37 ± 430	0.910
Zinc (mg)	12.46 ± 4.87	13.01 ± 4.73	0.54 ± 3.69	12.17 ± 4.38	12.26 ± 4.98	0.09 ± 3.94	0.544
Iron (mg)	13.26 ± 4.63	13.42 ± 4.86	0.17 ± 3.81	13.15 ± 4.74	12.56 ± 4.82	-0.59 ± 4.17	0.344
Potassium (mg)	3313 ± 1173	3642 ± 1174	329 ± 811*	3196 ± 1028	3091 ± 932	-106 ± 775	0.067
Phenylalanine (mg)	4239 ± 1462	4168 ± 1152	-70 ± 1005	3927 ± 1369	4117 ± 1410	190 ± 1288	0.572
Tryptophan (mg)	1121 ± 407	1100 ± 335	-21 ± 283	1041 ± 398	1088 ± 409	47 ± 360	0.816
Tyrosine (mg)	3390 ± 1244	3335 ± 1011	-55 ± 859	3143 ± 1135	3311 ± 1164	167 ± 1063	0.583
Isoleucine (mg)	4572 ± 1805	4432 ± 1427	-139 ± 1236	4198 ± 1618	4392 ± 1715	194 ± 1479	0.512
Leucine (mg)	7478 ± 2762	7389 ± 2226	-89 ± 1877	6955 ± 2466	7302 ± 2609	347 ± 2357	0.657
Valine (mg)	5248 ± 1917	5166 ± 1584	-82 ± 1323	4896 ± 1805	5097 ± 1888	201 ± 1645	0.712

Abbreviations: MFA, monounsaturated fatty acids; PFA, polyunsaturated fatty acids; SD, standard deviation; SFA, saturated fatty acids; [1]Intention to treat (ITT) population. The dietary supplements were not included in these data; *p*-Value: **, $p < 0.001$; *, $p < 0.05$ (Wilcoxon test within groups); [a] Mann-Whitney U test.
